# *Ebbie*: automated analysis and storage of small RNA cloning data using a dynamic web server

**DOI:** 10.1186/1471-2105-7-185

**Published:** 2006-04-03

**Authors:** H Alexander Ebhardt, Kay C Wiese, Peter J Unrau

**Affiliations:** 1 Department of Molecular Biology and Biochemistry, Simon Fraser University, 8888 University Drive, V5A 1S6 Burnaby, B.C., Canada; 2 School of Computing Science, Simon Fraser University Surrey, 13450 102nd Ave, V3T 5X3 Surrey, B.C., Canada

## Abstract

**Background:**

DNA sequencing is used ubiquitously: from deciphering genomes[1] to determining the primary sequence of small RNAs (smRNAs) [2-5]. The cloning of smRNAs is currently the most conventional method to determine the actual sequence of these important regulators of gene expression. Typical smRNA cloning projects involve the sequencing of hundreds to thousands of smRNA clones that are delimited at their 5' and 3' ends by fixed sequence regions. These primers result from the biochemical protocol used to isolate and convert the smRNA into clonable PCR products. Recently we completed a smRNA cloning project involving tobacco plants, where analysis was required for ~700 smRNA sequences[6]. Finding no easily accessible research tool to enter and analyze smRNA sequences we developed *Ebbie *to assist us with our study.

**Results:**

*Ebbie *is a semi-automated smRNA cloning data processing algorithm, which initially searches for any substring within a DNA sequencing text file, which is flanked by two constant strings. The substring, also termed smRNA or insert, is stored in a MySQL and BlastN database. These inserts are then compared using BlastN to locally installed databases allowing the rapid comparison of the insert to both the growing smRNA database and to other static sequence databases. Our laboratory used *Ebbie *to analyze scores of DNA sequencing data originating from an smRNA cloning project[6]. Through its built-in instant analysis of all inserts using BlastN, we were able to quickly identify 33 groups of smRNAs from ~700 database entries. This clustering allowed the easy identification of novel and highly expressed clusters of smRNAs. *Ebbie *is available under GNU GPL and currently implemented on

**Conclusion:**

*Ebbie *was designed for medium sized smRNA cloning projects with about 1,000 database entries [6-8].*Ebbie *can be used for any type of sequence analysis where two constant primer regions flank a sequence of interest. The reliable storage of inserts, and their annotation in a MySQL database, BlastN[9] comparison of new inserts to dynamic and static databases make it a powerful new tool in any laboratory using DNA sequencing. *Ebbie *also prevents manual mistakes during the excision process and speeds up annotation and data-entry. Once the server is installed locally, its access can be restricted to protect sensitive new DNA sequencing data. *Ebbie *was primarily designed for smRNA cloning projects, but can be applied to a variety of RNA and DNA cloning projects[2,3,10,11].

## Background

Small RNAs (smRNA) are currently of great interest, as they provide an additional and unanticipated level of gene control in higher eukaryotic organisms[12]. These smRNAs, 21–26 nt in length, act as guide sequences to specifically cleave or inhibit the translation of mRNA and also target the methylation of genomic DNA[13] and combat viral infection in certain organisms[14].

**Figure 1:**
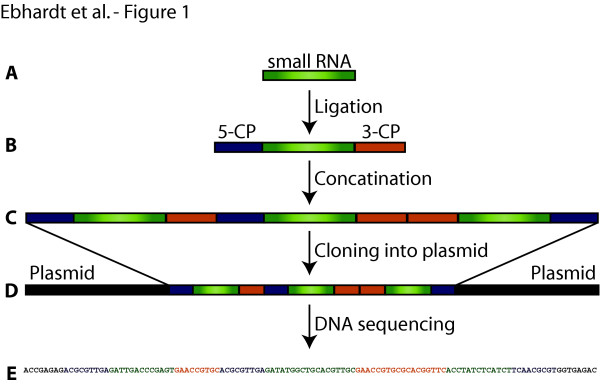
**Small RNA cloning strategy**. (A) SmRNAs are isolated by size from a sample of total RNA. (B) Then, 5'- and 3'-cloning primers (5-CP and 3-CP) are ligated to smRNAs using T4 RNA ligase[15]. Reverse transcriptase converts RNA to cDNA, which can be subsequently (C) concatenated. (D) These long strands of DNA are then ligated into a plasmid[29], transformed into bacteria and plasmids from single colonies isolated for DNA sequencing. (E) The resulting DNA sequence contains the original smRNA sequence flanked by two cloning primers (5-CP and 3-CP).

The first smRNAs were cloned from *C. elegans *in 2001[15] and ever since smRNA cloning proved to be a vital aspect of smRNA research. The process of smRNA cloning appends known primers to each end of the smRNA using T4 RNA ligase. The resulting constructs are then reverse transcribed and PCR amplified (Figure 1). Cloning and sequencing of these PCR products determines the full sequence of the cloned smRNA and is currently the most conventional approach that gives this information. Mainstream techniques such as microarrays[16] and Northern analysis[17] require that the primary sequence of smRNA be known. While, massively parallel signature sequencing can give estimates of total smRNA numbers[18], it does not provide primary sequence information. Bioinformatic approaches while useful are not currently able to accurately predict all known smRNAs and are unable to easily identify poorly conserved smRNAs sequences[2,19,20]. Thus, cloning is the most conventional technique currently available to reliably determine the sequence of expressed smRNAs.

Characterizing smRNAs from virally infected tobacco plants, Ebhardt *et al*.[6] discovered that smRNAs were modified on the 2'-hydroxyl of their 3' terminal ribose. This finding was made possible by a detailed comparison of the length of radiolabeled smRNAs with that observed after cloning and sequencing. The scores of resulting sequence files required an automated approach to efficiently uncover clusters of related sequences from both plant and viral genomes. Therefore, an online analysis pipeline called *Ebbie *was designed, which excises multiple instances of smRNA sequence from a DNA sequencing text-file, deposits the smRNA sequences into a MySQL database and performs BlastN searches of these inserts against various databases.

## Implementation

### External software

Blast v2.2.9 is a heuristic local alignment tool essential for comparing query sequences to large databases[9]. When installed locally, it proves to be a powerful and versatile tool for comparing new sequences to personalized local databases. For our published study, a blast-database containing 1,919 smRNA sequences (43,724 nucleotides) was installed locally. Querying this database using BlastN, overlaps of at least 8 consecutive base pairs were detectable using default parameters. This was sufficient for our cloning project of ~700 smRNA clones. For larger databases, optimized Blast parameters might be necessary. If genomic sequence data is available, BLAT[21] might be considered for annotating smRNAs to the genome.

### Components of *Ebbie*

MySQL v4.1.10a-Max was chosen as a database due to its compatibility with Perl. Perl v5.6.0 was chosen as a programming language because of its strength in analyzing and manipulating strings[22]. Perl serves well in creating dynamic web pages[23], interacting with MySQL databases[24] and communicating with the operating system. Most Linux systems are distributed with these programs. *Ebbie *was implemented on a standard PC with Linux Novell SuSE 9.3 operating system (standard PC: AMD Athlon 1.1 GHz processor with 256 KB cache, 512 MB RAM, 60 GB HD) and a RedHat Linux apache2 server. Installation notes are provided in the supplement [see Additional file 1].

### Flowchart of *Ebbie*

**Figure 2:**
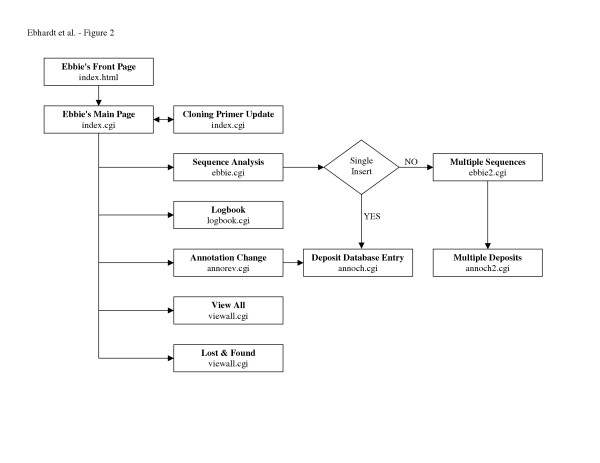
**Flowchart of *Ebbie***. Indicated are *Ebbie*'s functions and the cgi-scripts required for each. *Ebbie*'s front page is created by index.html. The database information is submitted to *Ebbie*'s main page, created by index.cgi. Index.cgi can update the currently used cloning primers (5-CP and 3-CP). From the main page, several scripts can be called. DNA sequencing text files are uploaded and analyzed by ebbie.cgi. Depending on the number of inserts found, annoch.cgi is called to deposit the insert into the MySQL database. If multiple inserts are found, ebbie2.cgi and annoch2.cgi are used for the same purpose. From *Ebbie*'s main page a range of database review/manipulation tools can be accessed: *Logbook *(table created by logbook.cgi), *View All*, *Lost & Found *(tables created by viewall.cgi) and *Annotation Change *(also using annoch.cgi to update database entry).

*Ebbie *is comprised of eight perl-cgi-scripts. They share four common libraries, which contain common subroutines, e.g. querying the MySQL database and drawing a table with the results. Figure 2 shows *Ebbie*'s functions and the perl-cgi scripts required for each. *Ebbie*'s front page is created by index.html. The database information is submitted to index.cgi, *Ebbie*'s main page. Index.cgi can update the currently used cloning primers (5'-cloning primer: 5-CP and 3'-cloning primer: 3-CP). From the main page, several scripts can be called. The *Logbook *lists all analyzed files (table created by logbook.cgi). DNA sequencing text files are uploaded and analyzed by ebbie.cgi when *Sequence Analysis *is selected. If a single insert is found, annoch.cgi is called to deposit the insert into the MySQL database. If multiple inserts are found, ebbie2.cgi and annoch2.cgi are used to add these entries to the MySQL database. From *Ebbie*'s main page a range of database review/manipulation tools can also be accessed: *View All, Lost & Found *(tables created by viewall.cgi) and *Annotation Change *(which also uses annoch.cgi to update a database entry). For a detailed discussion of these functions see text below.

## Results and discussion

### Description of *Ebbie*

**Figure 3:**
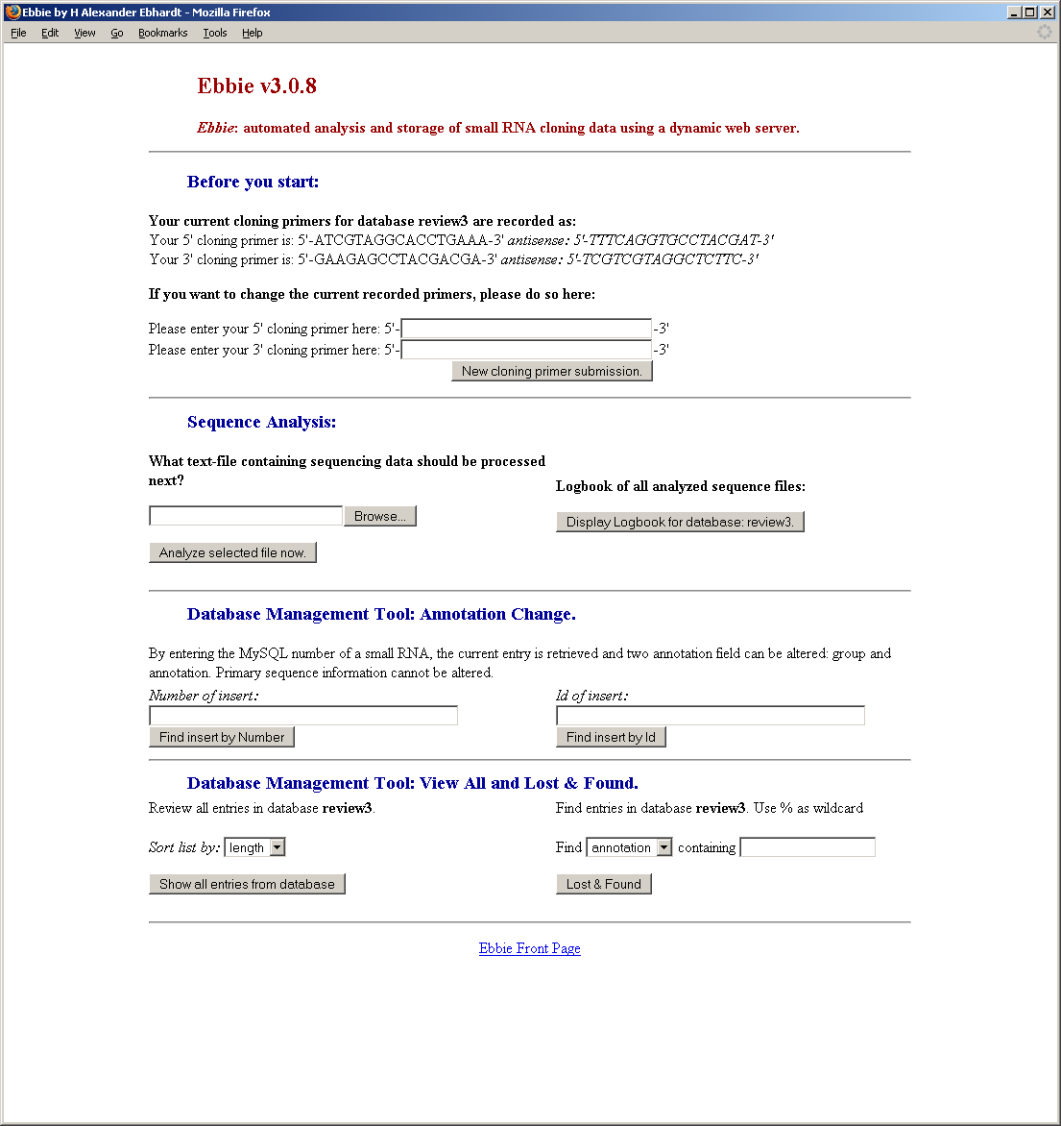
**Main page of *Ebbie***. The user is able to (1) review the current cloning primer pairs and update them if necessary. (2) Sequencing file analysis by uploading a text file containing the sequencing data and performing a series of BlastN searches; the cloned inserts are deposited into the selected database. A logbook records the analyzed files, the time of analysis, the number of insert(s) within the sequencing file or an error message if the sequencing file cannot be correctly analyzed. (3) Database management tool: *Annotation Change*. A database entry is retrieved and annotations can be updated. (4) Database management tools: *View All *and *Lost & Found*. All database entries are displayed, sorted by various parameters

*Ebbie*'s dynamic web pages are platform and browser independent (browsers tested: Mozilla Firefox 1.0.4 on Linux and Windows XP operating system, Safari 2.0 for Macintosh, MS Internet Explorer 6.0 on Windows XP). A tutorial for *Ebbie *is also available in the online supplements [see Additional file 2]. The front page requests the user to select a particular database before entering the program. Once selected, *Ebbie *subsequently works with this database unless the database selection is changed on the front page. As seen in Figure 3, *Ebbie*'s main page has four basic functions. First, it retrieves the current 5'- and 3'-cloning primers (5-CP, 3-CP) from the selected MySQL database and displays their sense and antisense sequence. The user can edit these sequences as desired. All sequences entered into these text fields are capitalized. Second, the user can browse the local computer's file manager to select and analyze a DNA sequencing text file. *Ebbie *maintains a log of all processed files that can be reviewed by clicking on the *Display logbook *function. Through *Ebbie*'s main page, three online database management tools are available: *Annotation Change*, *View All *entries of database and *Lost & Found*.

The uploaded file name serves as the primary *id *for the MySQL database entry. If no file is selected or if the *id/filename *already exists in the database, an error message is displayed and the process aborted. If a file is valid (i.e. it is new and unique), the DNA sequence data is converted into a string, capitalizing the characters A, C, G and T. All other characters remain unchanged. Perl's *index *function is used to confirm that at least one 5-CP and 3-CP pair exists, if this condition is not met or if perl's *index *function identifies an uneven number of 5-CP and 3-CP pairs, then an appropriate error message is generated in the logbook. The algorithm starts at the 5' end of the DNA sequence and finds the first occurrence of a 5-CP (or antisense 3-CP). Moving in the 3' direction, the next 3-CP (or antisense 5-CP) is located. An insert is deposited into the MySQL database, if a sequence of length > 0 is found between the two primer pairs. If no insert is found in the DNA sequencing text-file, a message is displayed and recorded in the logbook.

### Database selection

On the front page of *Ebbie*, the user can choose between different databases. These database names correspond to the names used to setup the MySQL database on a given implementation of *Ebbie*. These databases can be customized by editing the file/ebbie/lib/mysql.lib#sub:mysqldb and *Ebbie*'s front page (index.html). Once a database is selected from the front page of *Ebbie*, the user will work with the chosen database until another database is selected by returning to the front page. BlastN flat files are kept for each database in order to allow continually updated BlastN comparison with the growing MySQL database.

The *'Database Management Tool: Annotation Change' *allows the user to change only two fields of each insert: '*annotation*' and '*group*', all other fields (*no, id, sequence, length, orientation *and *sample source*) cannot be edited in order to preserve the integrity of the database. This restriction was deliberately chosen to maximize the integrity of primary data.

### Analysis of inserts

Once an insert is found, *Ebbie*:

- Deposits the *id *and sequence insert into the dynamic BlastN database,

- Deposits the insert into the MySQL database, in the correct sense specified by the orientation of 5-CP and 3-CP,

- Determines the inserts *length*,

- Determines its *id *based on the file name, and

- Determines its *sample source*, which is inferred from the first character of the file name.

The last function relies on *grep *to determine the initial character and then assigns the sample source by referencing an external text file. This sample source assignment can easily be manipulated by editing the external text file (/ebbie/mod/source.nt). Currently, file names starting with 1, 2, ... 9 have an automatic sample source assigned; other file names will result in '*unknown*' sample source.

**Figure 4:**
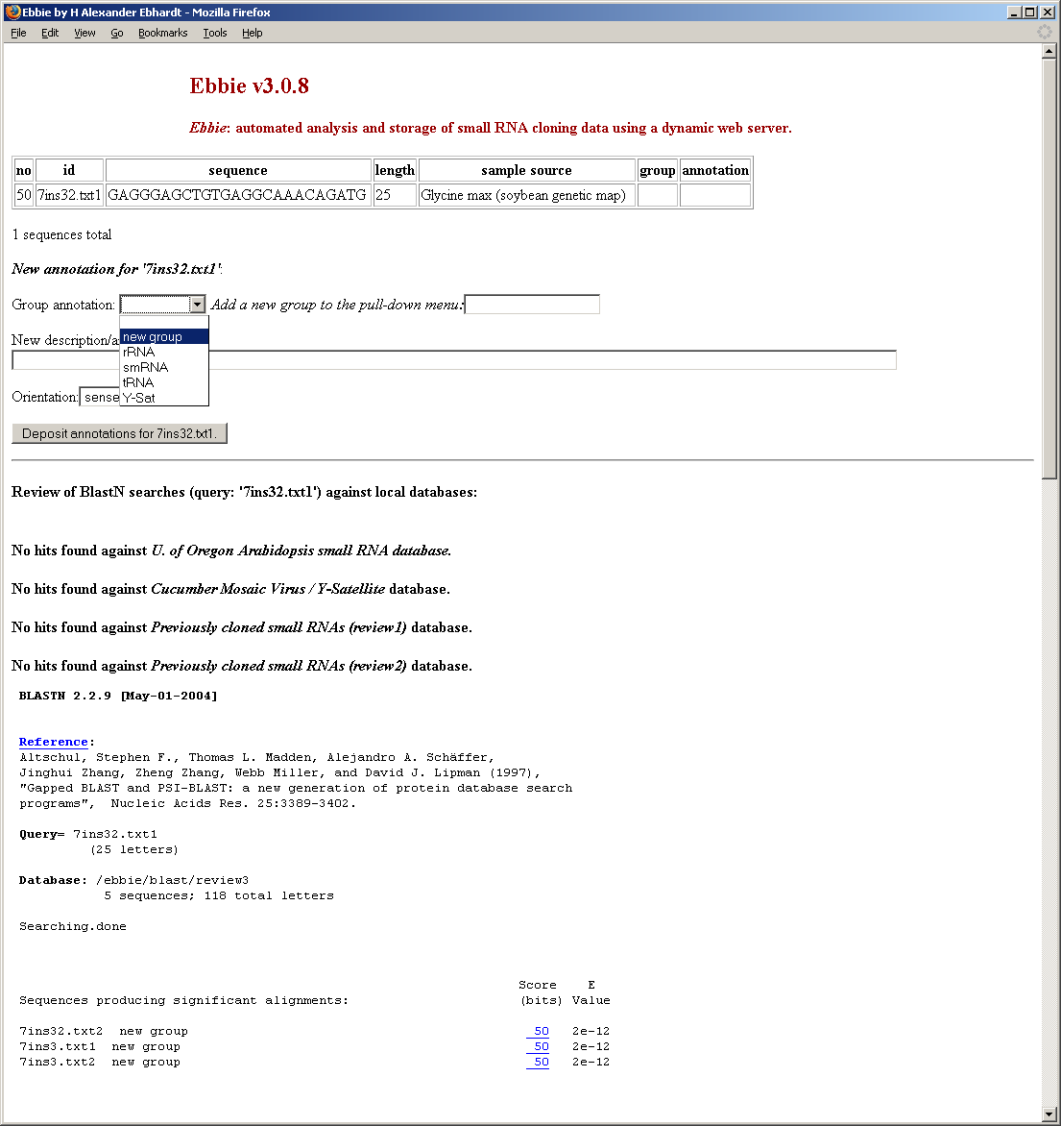
**Analyzing a cloned smRNA**. The first table shows the MySQL database entry, which is created automatically once a cloned smRNA is found (entering *id*/filename, sequence of smRNA in correct 5'-3' orientation, *length *and *sample source*). The *group *annotation pull-down menu offers the user a choice from a variety of *group *annotations. A new *group *can be named in the adjacent text field (it will be entered into the MySQL entry of the clone in question and the pull down menu will be updated and alphabetically sorted with the new *group*). Further *annotation *details can be added in the *annotation *text field. The *orientation *field offers three choices: N/A, antisense and sense.

Following the automated sequence deposition, the sequence insert is subject to BlastN searches against locally installed databases. In our case, the BlastN searches included the BlastN database from the *Arabidopsis *smRNA-cloning project[25], the genomes of Y-Satellite plus its helper virus Cucumber Mosaic Virus (NCBI accession numbers for viral genomes: NC_002034.1, NC_002035.1, NC_001440.1, D10038.1) and a complete BlastN database of all previously found inserts. The latter dynamic BlastN database extends each time a new insert is found, allowing for rapid identification of new groups. Each BlastN analysis is scanned by *grep*, probing for '*No hits found*.' in which case it will only print one line onto the screen, indicating an unsuccessfully searched database. Otherwise, the complete BlastN analysis is displayed on the web page to facilitate user-mediated annotation. Figure 4 shows the analysis of a clone, in this case finding a complete match against three previous entries.

The user can now fill out three additional annotation fields:

(1) *Group *pull-down menu: The *group *pull-down menu offers standard RNA types found previously during data entry and analysis. A *new group *can be added through the accompanying text field if a *group *is identified by local BlastN analysis. Once submitted, this *new group *description is simultaneously added to the smRNA annotation in the MySQL database, the BlastN dynamic database and the *group *pull-down menu. The latter menu is sorted alphabetically and is made available for subsequent *group *annotations. This form of annotation proved quite powerful in the analysis of our set of smRNAs.

(2) *Annotation *field: a text field allowing for user generated comments based on the automatic BlastN searches or external BlastN searches (our BlastN searches were limited by the amount of RAM available on the server).

(3) *Orientation *pull-down menu: allows the selection of three categories: N/A, sense and antisense to classify the BlastN search results. This is important when working with smRNAs as smRNAs are known to be produced by RNA dependent RNA polymerases that synthesize the reverse complement of their original genomic sequence[26].

Once annotated, the insert's MySQL entry is updated by pressing the submit button. Consecutively, *Ebbie*'s deposit algorithm appends the *id*, *group *annotation (if applicable) and insert sequence in FASTA format into a BlastN flat file. The flat file is subsequently formatted for subsequent BlastN analysis. The newly created web page displays the MySQL entry (*id*, *sequence*, *length*, *group *and *annotation*) and allows the user to return to *Ebbie*'s main page.

An example: rRNA group 01

During our smRNA cloning project of virally infected tobacco plants[6], *Ebbie *identified 33 groups among 700 smRNA sequences. (We classified a group as two sequences overlapped by 12 or more consecutive base pairs. This empirical overlap proved to be stringent in retrospect; a 16 base pair non-gapped overlap would have resulted in 32 groups. A percentage overlap was not chosen, as a BlastN search might not align the whole query sequence to any given subject, thus misleading the user about the percentage identity.) The first group *Ebbie *identified in infected/non-infected tobacco plants was a 24 nt long smRNA resulting from the end of the small ribosomal RNA. This accumulation is an intriguing fact and does not seem random, considering that 18S rRNA is approximately 1,800 nts in length. Currently, this group is under further investigation.

### Multiple inserts

If the number of inserts in the sequencing file exceeds one, all inserts are automatically entered into the MySQL database in the correct 5'-3' orientation, together with their *length *and *sample source*. The *id *for each insert is specified uniquely by appending to the end of the filename a unique insert number. The user is notified of the number of primer pairs found and the number of inserts deposited into the MySQL database. To analyze individual sequences, a pull-down menu is created that displays all inserts found in the current sequencing file. Following the selection of an insert, the user can analyze each one individually (as described in the previous section above). As long as unannotated inserts are in the database, the user can select from the pull-down menu the inserts that remain to be annotated.

### Logbook function

The logbook function is reached from *Ebbie*'s main page. Each time a file is uploaded and analyzed by *Ebbie*, the system time is recorded, together with the filename. Once the file is analyzed, a comment is recorded depending on the outcome of the analysis: 'Sorry, there was no insert found', 'Single insert found.', 'There were *x *primer pairs and *y *inserts deposited into *z*' (where *x *is the number of primer pairs found, *y *the number of inserts deposited and *z *the database used) and 'Number of 5'- and 3'-cloning primers not even!'. The last comment is displayed in red, as this file may need manual intervention to rescue its content before subjecting it again to the insert excision algorithm.

### Review database

All entries in the selected database can be reviewed and ordered by *id*, *length*, *group *and *number *fields using the '*View All*' button. For each database, *Ebbie *will remember the last selection of this pull-down menu. This feature is useful while generating a database and allows a quick survey of the database during data entry.

### Lost & found

The *Lost & Found *function allows the user to use one or more wildcard characters for querying the database. '_' is used for single character and '%' for multiple character wildcard. From a pull-down menu the user selects a category, e.g. *id*, and in the adjacent text field the query is entered, e.g. '3%'. In this example, all entries with the starting character of '3' would be displayed.

For more complex queries, a second pull-down menu is available, which includes AND/OR BOOLEAN operators. For example, all smRNAs belonging to the class of "Y-Sat RNA" AND length of "21" nucleotides can be selected.

### Change annotation

To update the *annotation *of individual inserts, a *change annotation *script was implemented. The script searches for either the *id *or *number *of the insert. The *id *is useful once a new *group *is identified in, for example, a BlastN search result. The *number *is convenient after reviewing the database. Once a *number *or *id *has been submitted, the record of the *id *is retrieved from the database (*no*, *id*, *sequence*, *length*, *sample source*, *group *and *annotation*). The user can then choose a standard *group *description from the *group *pull-down menu or add a *new group*. The *'Annotation field' *will display the current annotation in the text field, allowing the user to add supplementary information to it. Once adjustments are made, the new *annotation *can be submitted and the corresponding fields in the MySQL database are updated. Further, if the *group *annotation is changed, the BlastN flat file will be edited to reflect the current *group *annotation. The user is unable to use a wild card character for the *change annotation *function.

### Limitations of *Ebbie*

The algorithm will experience difficulties if low complexity or ambiguous repetitive 5-CP or 3-CPs primer sequences are used, which should be avoided by the correct design of primer pairs. Similar fundamental problems are encountered when cloning RNA using poly(A)-polymerase to extend the 3' end of a sequence which may already contain poly(A) residues. Additional wet lab experiments (e.g. primer extension assays) need to be conducted in order to determine the RNA's true 3' end/length. Also, no wild card characters are allowed when identifying 5-CP and 3-CP primers within the DNA sequence file, to ensure the quality of the DNA read. Imperfect primers can be identified by a subsequent manual examination of sequence files that are flagged as having uneven or no primer pairs in the logbook.

### Comparable software

To our knowledge, no comparable software exists. Other DNA sequencing programs are concerned with automated base calling, e.g. *phred*[27,28]. The closest DNA sequence analysis tools are vector-trimming programs, which remove external vector sequences from the DNA sequence. In the case of single inserts, this kind of algorithm could be used, but it would still leave the insert surrounded by the 5-CP and 3-CP primers. Also, once the vector is removed, there is typically no further analysis of the remaining sequence, e.g. BlastN search. For multiple inserts, vector removal programs are unsuitable, as they would result in a single insert consisting of a concatenated set of inserts flanked by 5-CP and 3-CPs.

### Future directions

Besides local BlastN searches, it is also feasible to perform remote BlastN searches using NCBI's *netblast*. The web server (apache2) requires modification by setting the 'KeepAliveTimeout' to at least 200 seconds. Typically, this was the time interval necessary for *netblast *to return a BlastN search result and slowed down annotation time significantly. Some laboratories with a faster link to NCBI might consider this option for searching very large databases.

Currently, *Ebbie *analyzes DNA sequencing text files. *Ebbie *could be expanded using other DNA sequencing analysis software, e.g. base calling software *phred*. The latter software is not yet available under GNU GPL and was therefore not implemented in this version of *Ebbie*.

For cloning smRNAs it is desirable to display the length distribution of all or groups of smRNAs in a histogram. This function will be implemented in the near future.

## Conclusion

*Ebbie *is a semi-automated smRNA cloning data processing algorithm, which initially searches for any substring within a DNA sequencing text file, which is flanked by two constant strings. The substring, also termed smRNA or insert, is stored in a MySQL and BlastN database. The latter feature allows for rapid identification of high occurrence smRNAs. Our laboratory successfully used *Ebbie *to analyze scores of DNA sequencing data originating from a smRNA cloning project.*Ebbie*'s strength lies in the rapid annotation of sequences using locally installed BlastN, finding sets of smRNA clusters, reliable storage of valuable sequencing data and in eliminating manual mistakes during the excision process.

*Ebbie *is able to identify single and multiple inserts and is comprised of eight perl-cgi-scripts that use common subroutine libraries. External files allow other research groups to customize *Ebbie*, e.g. automatic *sample source *assignment is based on an external file, which is easily modified. Once *Ebbie *is installed on a local server, access can be restricted to allow for confidential DNA sequencing analysis. Installation notes are provided in the supplement [see Additional file 1]. Besides cloning of smRNAs, *Ebbie *can be used for any type of sequence analysis where two constant regions flank the sequence of interest. The reliable storage of annotated inserts in a MySQL database, instant BlastN analysis of new inserts to previously installed databases and previous inserts make it a powerful new tool in any laboratory using DNA sequencing[2,3,6-8,10,11].

## Availability and requirements

**Project home page: **

**Operating system(s): **developed on Linux, Suse 9.3; suitable for LINUX, UNIX, Mac

**Programming languages: **Perl (Perl modules: -mCGI, -mDBI), MySQL, html

**Other requirements: **Safari 2.0 or higher, Firefox 1.0.3 or higher

**License: **GNU GPL

**Any restrictions to use by non-academics: **GNU GPL

## Abbreviations

5-CP: 5' cloning primer

3-CP: 3' cloning primer

BLAST: Basic Local Alignment Search Tool

GNU GPL: General Public License

NCBI: National Center for Biotechnology Information

MySQL: SQL: Standard Querying Language

mRNA: messenger RNA

nt: nucleotide

PCR: polymerase chain reaction

rRNA: ribosomal RNA

tRNA: transfer RNA

## Authors' contributions

HAE had the initial idea for this project, did all coding (perl, html and mysql), wrote the manuscript and cover letter. KCW substantially revised the initial idea and guided the early development on *Ebbie*. PJU broadened the scope of *Ebbie*, thus it is now a versatile tool to analyze DNA sequencing data. KCW and PJU reviewed the manuscript critically and improved it to target a wider audience. All authors read and approved the final manuscript.

## Supplementary Material

Additional File 1 Installation Notes for *Ebbie*. Installation notes for installing *Ebbie *on a Linux server. Click here for file

Additional File 2 Tutorial of *Ebbie*. provides step-by-step guide to *Ebbie v 3.0.8*. Click here for file
