# Supplementary material for: Ebbie: automated analysis and storage of small RNA cloning data using a dynamic web server
Source: BMC Bioinformatics. 2006 Apr 3;7:185. doi: 10.1186/1471-2105-7-185 (PMC1450305; doi:10.1186/1471-2105-7-185)
Supplement: Additional File 1 — Installation Notes for Ebbie. Installation notes for installing Ebbie on a Linux server. [file 1471-2105-7-185-S1.pdf]

Copy Ebbie folder onto harddrive

The folder /ebbie/ contains various subfolders:

Folder name	Main function	Notes
/ebbie/blast	Blast and blast related files, including blast searchable databases	- once Blast2.2.9 downloaded, adjust blastall and formatdb directory structure in /ebbie/lib/blast.lib
/ebbie/examples	Various examples to test the algorithm	
/ebbie/lib	Contains all perl libraries	- html.lib: please change all current URLs against your own URLs
/ebbie/log	Contains logbooks	
/ebbie/mod	Contains modules for html page	- source.nt: each line can be replaced with your own sample description, e.g. if your file name starts with '3', then the 3rd line in this file will be used to annotate the sample source. Edit with basic editor, such as pico, vi or emacs<br>- groups.nt: pull-down menu for group annotation can be edited from web page or here with pico, vi or emacs

Setting up Apache2 on Linux (SuSE).

The apache2 server was activated and the active firewall was modified to allow http traffic to the server.

For Authorization and Access Control read: http://httpd.apache.org/docs/2.0/howto/auth.html

OR, one example to restrict access with '.htpasswd':
To increase security, all access to the server below the /src/www/htdocs directory was disallowed in the /etc/apache2/httpd.conf file:

```
<Directory />
Options None
AllowOverride AuthConfig
Order deny,allow
Deny from all
</Directory>
```

Restricting access to the web server by:

```
/srv/www/htdocs/.htpasswd:
AuthType Basic
AuthUserFile /srv/www/.htpasswd
AuthName "enter login/passwd"
require valid-user
```

Also, these .ht* files had to be protected from public viewing by adding to /etc/apache2/httpd.conf file:

```
<Files ~ "^\.ht">
Order allow,deny
Deny from all
</Files>
```

Setting up users and user passwords with .htpasswd:

/usr/sbin # ./htpasswd2 -b /srv/www/.htpasswd *user password*

According to the online documentation of apache2 server, running dynamic programming scripts requires the activation of the cgi-bin folder in the /etc/apache2/httpd.conf file as follows:

```
<Directory /srv/www/cgi-bin>
Options +ExecCGI
AddHandler cgi-script .cgi
</Directory>
```

Setting up CGI scripts

confirm that your perl is in the same directory than these scripts (first line of all cgi-scripts have to be adjusted if perl's directory is not: '/usr/bin/perl' = to find out where your perl is installed type into command line: 'which perl')

For more info on activating the apache2-cgi server go to: http://httpd.apache.org/docs/2.0/howto/cgi.html

Setting up Blast

- download blast 2.2.9 form ftp://ftp.ncbi.nlm.nih.gov/blast/executables/release/2.2.9/
- decomress file: tar -zvvf blastfile
- download and format nt-databases included in this distribution for blast searches e.g. ./formatdb -i asrp.nt -p F -o T
- make sure all files and /ebbie/blast directory are 777 (chmod 777 *)
- check for correct path to your blast distribution in library /ebbie/lib/blast.lib

Setting up MySQL

$mysqldb = is database of your choice, here the database is called 'review1'.

1) log in as superuser to mysql, e.g. mysql -u root -p ↲

2) mysql> create database review1; ↲

```
Query OK, 1 row affected (0.00 sec)
```

3) mysql> use review1; ↲

```
Database changed.
```

4) mysql> grant select, insert, update on review1.* to T100@localhost; ↲

```
Query OK, 0 rows affected (0.03 sec)
```

5) mysql> create table review1(↲
-> no smallint(6) not null auto_increment, ↲
-> id varchar(20) not null, ↲
-> sequence varchar(50) not null, ↲
-> class varchar(40), ↲ // Note: 'group' is a MySQL command, can not be used as column name
-> length smallint(6), ↲
-> source varchar(40) not null, ↲
-> orientation varchar(20), ↲
-> description varchar(255), ↲
-> date timestamp(14), ↲
-> primary key(no)); ↲

```
Query OK, 0 rows affected (0.09 sec)
```

6) mysql> describe review1; ↲

```
+-------------+--------------+------+-----+-------------------+----------------+
| Field       | Type         | Null | Key | Default           | Extra          |
+-------------+--------------+------+-----+-------------------+----------------+
| no          | smallint(6)  |      | PRI | NULL              | auto_increment |
| id          | varchar(20)  |      |     |                   |                |
| sequence    | varchar(50)  |      |     |                   |                |
| class       | varchar(40)  | YES  |     | NULL              |                |
| length      | smallint(6)  |      |     | 0                 |                |
| source      | varchar(40)  | YES  |     | NULL              |                |
| orientation | varchar(20)  | YES  |     | NULL              |                |
| description | varchar(255) | YES  |     | NULL              |                |
| date        | timestamp    | YES  |     | CURRENT_TIMESTAMP |                |
+-------------+--------------+------+-----+-------------------+----------------+
9 rows in set (0.00 sec)
```

7) mysql> grant insert,select,update on review1.* to 'T100'@'localhost';
Query OK, 0 rows affected (0.01 sec)

MySQL database + cgi-scripts
- make sure the database name is reflected in two scripts: index.html and /ebbie/lib/mysql.lib#sub:mysqldb

Populate MySQL database with cloning primer set
- log on to mysql command line monitor, e.g. mysql -u T100 review1 ↲
- The cloning primer selection on Ebbie's Main page relies on two entries called: 'DDRFDJ5' and 'DDRFDJ3'. Therefore, these ids have to be in the database. Add them by using the following insert command:

insert into review1 (id,orientation) values ('DDRFDJ3','sense'); ↲
insert into review1 (id,orientation) values ('DDRFDJ5','sense'); ↲

Done. The values of the cloning primer can be added through the Main Page of Ebbie. They are unique to each database installed (e.g. this process has to be repeated for each database).

mysql.lib: insert new db name
logbook: chmod 777

External files: groups for databases, sample source file etc.

/ebbie/mod/source.nt
grep determines the sample source by determining the first character of the sequencing filename. If the filename starts with '3' then *Ebbie* will assign whatever is written in the third line of **source.nt** (currently 'Lycopersicon esculentum'). This file can be customized by using a simple command line editor such as *pico, vi* or *emacs*.

/ebbie/mod/groups_review1
This file contains all groups for the standardized pull-down menu; group annotations are unique to each database.

/ebbie/mod/pulldown_ori
This pull-down menu file contains all options for the pull-down menu option for View All.

/ebbie/mod/pulldown_review1
This pull-down menu file contains all options for the pull-down menu option for View All. Each database has its own file to "remember" the last selection made.
