# Supplementary material for: Ebbie: automated analysis and storage of small RNA cloning data using a dynamic web server
Source: BMC Bioinformatics. 2006 Apr 3;7:185. doi: 10.1186/1471-2105-7-185 (PMC1450305; doi:10.1186/1471-2105-7-185)
Supplement: Additional File 2 — Tutorial of Ebbie. provides step-by-step guide to Ebbie v 3.0.8. [file 1471-2105-7-185-S2.pdf]

Ebbie v3.0.8

Ebbie: automated analysis and storage of small RNA cloning data using a dynamic web server.

(GNU GPL) by H Alexander Ebhardt

Tutorial

Ebbie v3.0.8

***Ebbie*: automated analysis and storage of small RNA cloning data using a dynamic web server.**

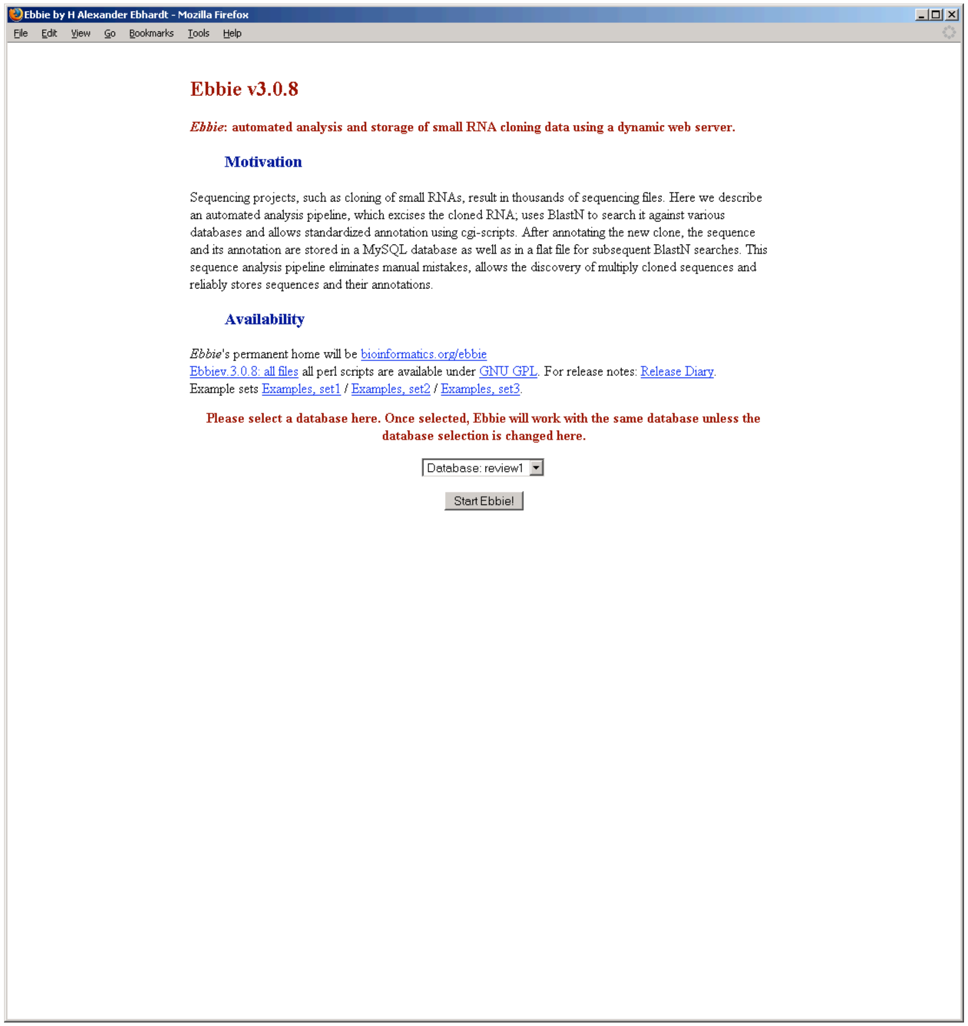

Front page of *Ebbie*.

- Links to
  - http://bioinformatics.org/ebbie
  - All files, GNU GPL, Release Diary
  - Example files

  can be found.
- Choose database here; *Ebbie* will use this database until it is changed on the front page.

Ebbie v3.0.8

Ebbie: automated analysis and storage of small RNA cloning data using a dynamic web server.

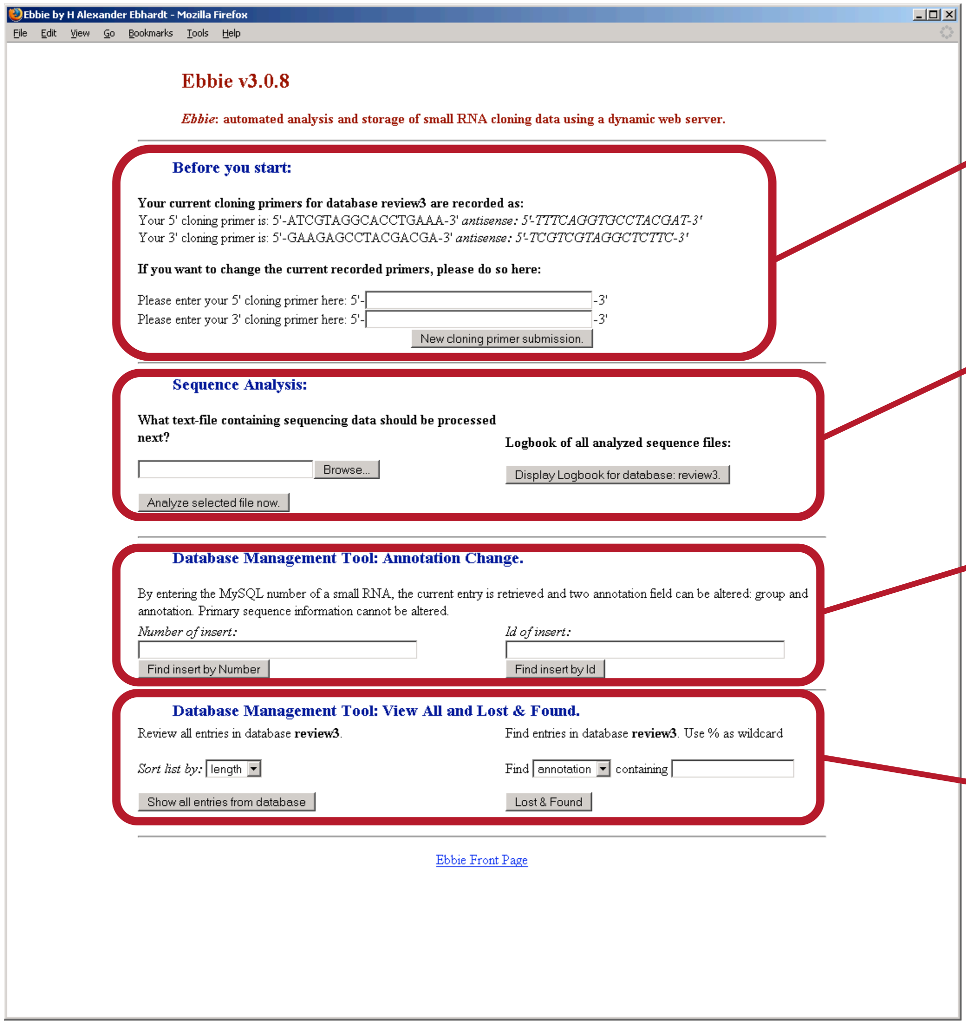

Main page of *Ebbie*.

- Before you start:
  - Reports 5′- and 3′-cloning primers (5-CP, 3-CP).
  - New primer pairs can be entered.
  - Only ACGT are valid characters.
- Sequence Analysis:
  - Selection of a text file containing DNA sequencing data.
  - Logbook of all files analyzed.
- Database Management Tool: Annotation Change
  - Search for insert by its database number (e.g. cloned small RNA).
  - Copy/Paste Id of insert (e.g. from BlastN search result).
- Database Management Tool: View All and Lost & Found
  - Review all entries, order by id, length, number or group.
  - Find entries by using wild card characters.

Ebbie v3.0.8

***Ebbie*: automated analysis and storage of small RNA cloning data using a dynamic web server.**

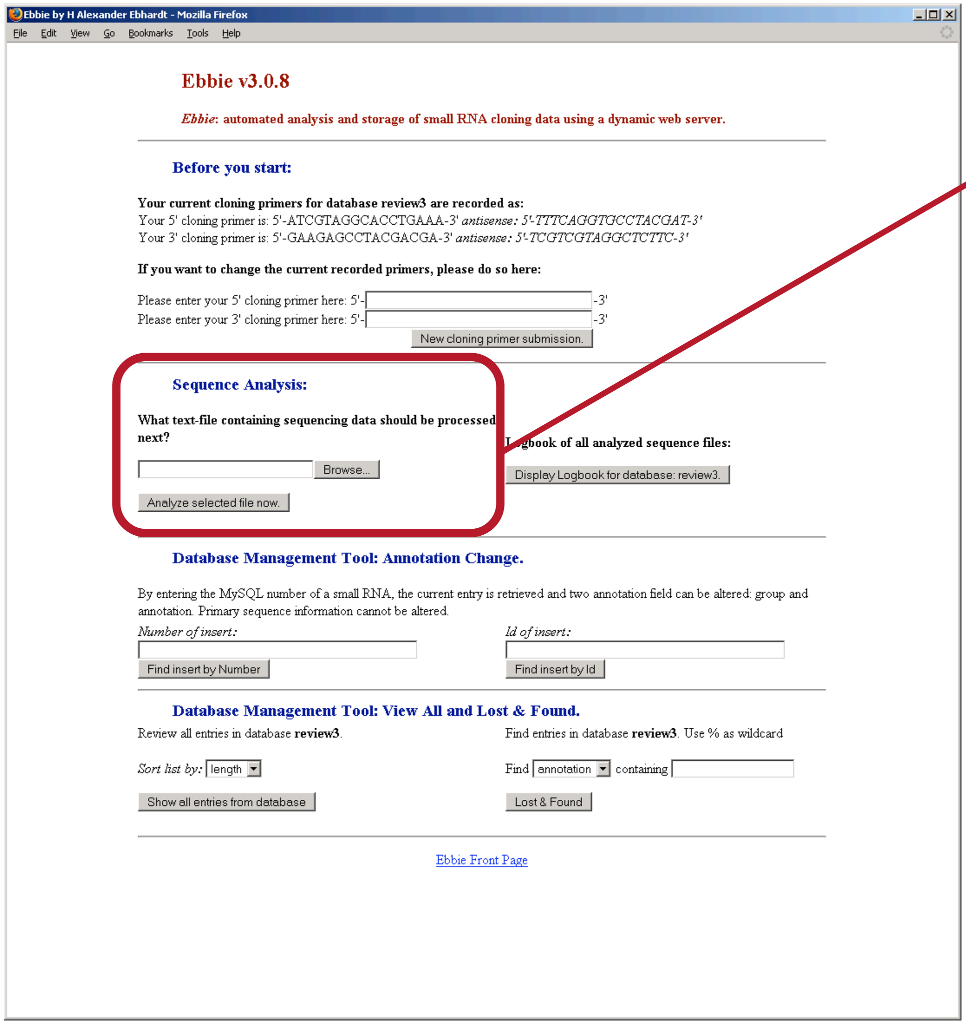

Main page of *Ebbie*.

- Sequence Analysis:
  - Uploading text file containing DNA sequencing data.

Ebbie v3.0.8

Ebbie: automated analysis and storage of small RNA cloning data using a dynamic web server.

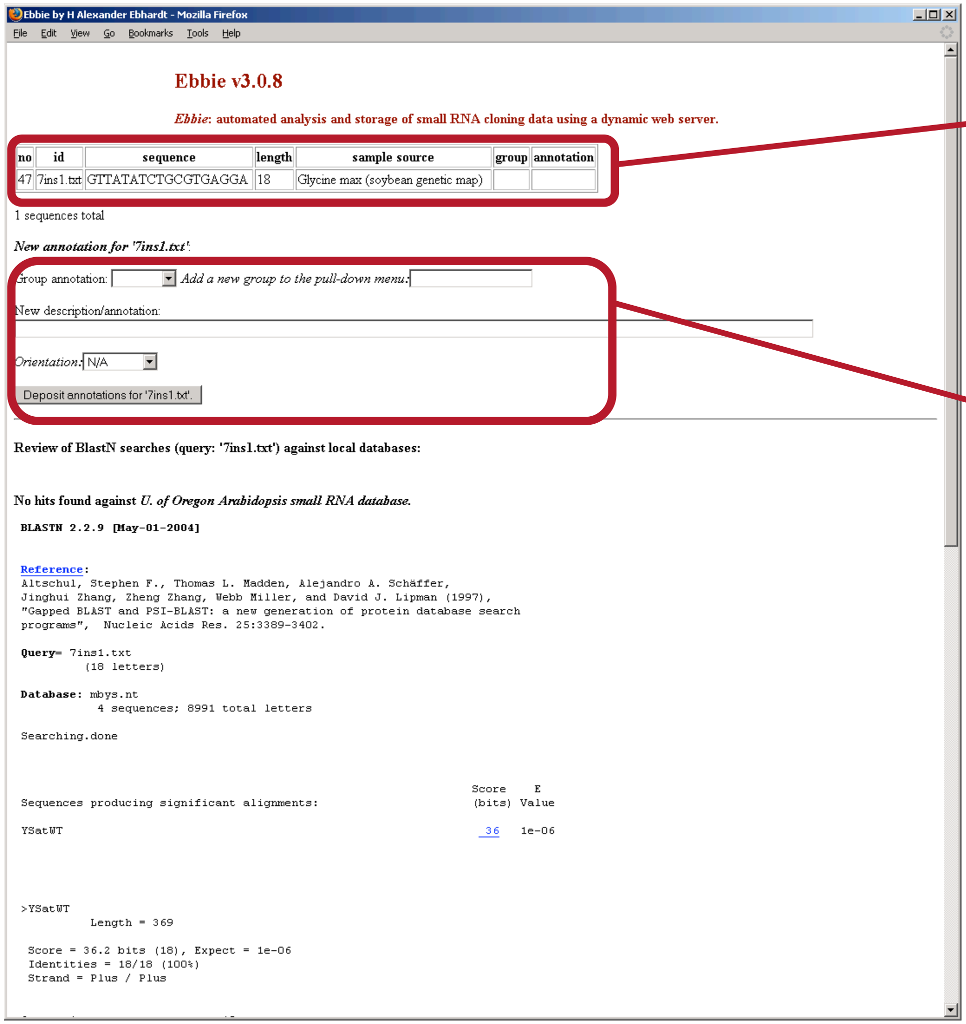

Ebbie: analyzing single insert

- Automatically deposits insert into MySQL:
  - Number (no, automatically)
  - Id (from filename)
  - Insert (nt sequence)
  - Length (counted insert)
  - Sample source (inferred from filename)
- Annotation fields:
  - Group annotation
    - Pull down menu
    - Add to pull down menu in text field
  - Additional comments
  - Orientation (N/A, sense, antisense)
- BlastN search results
  - Searches *Ebbie* associated databases.
    - If negative search result, then only 1 line displayed.
    - Otherwise, whole BlastN search result printed.
- Press ‘Deposit annotation for … ‘ to update insert annotation and add to BlastN database.

Ebbie v3.0.8

Ebbie: automated analysis and storage of small RNA cloning data using a dynamic web server.

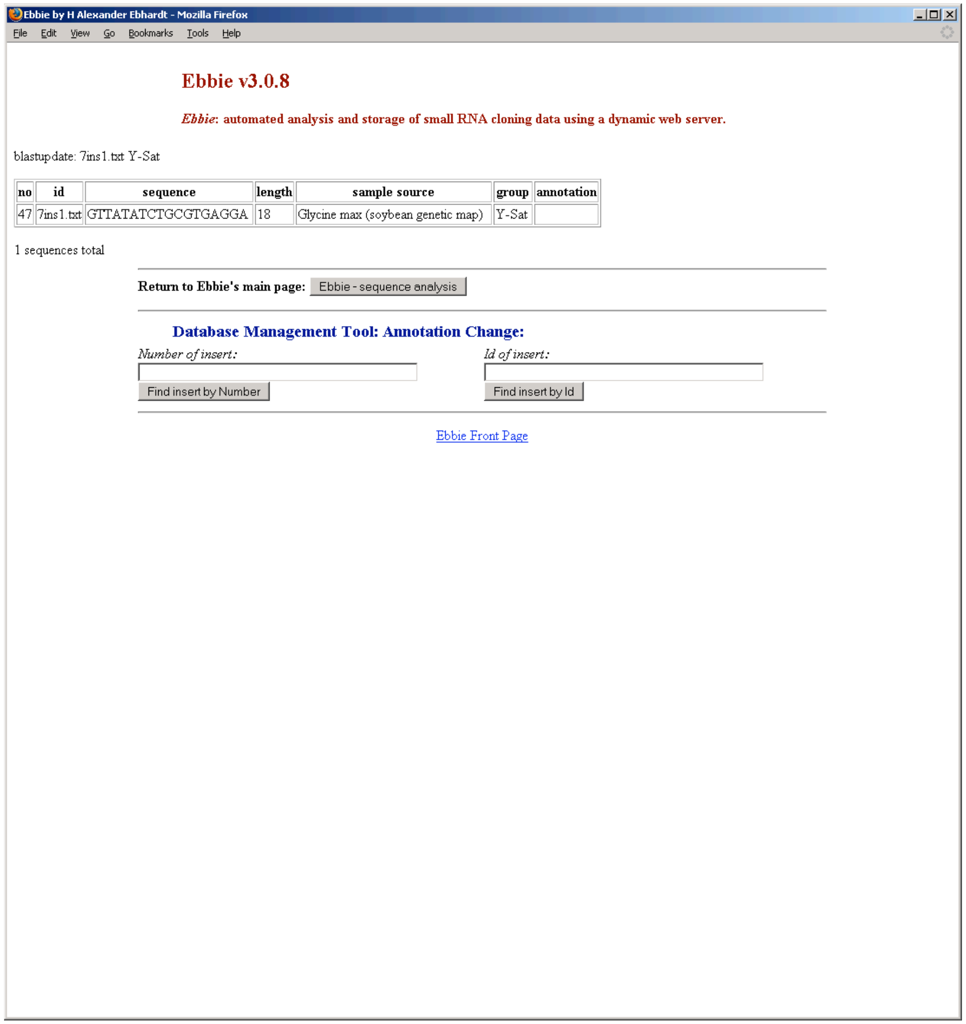

Ebbie: depositing single insert

- – BlastN id and group displayed.
- – MySQL entry displayed.

- Return to *Ebbie*'s Main Page for more sequence analysis
- OR: enter number or id of insert for Database Management Tool: Annotation Change

Ebbie v3.0.8

Ebbie: automated analysis and storage of small RNA cloning data using a dynamic web server.

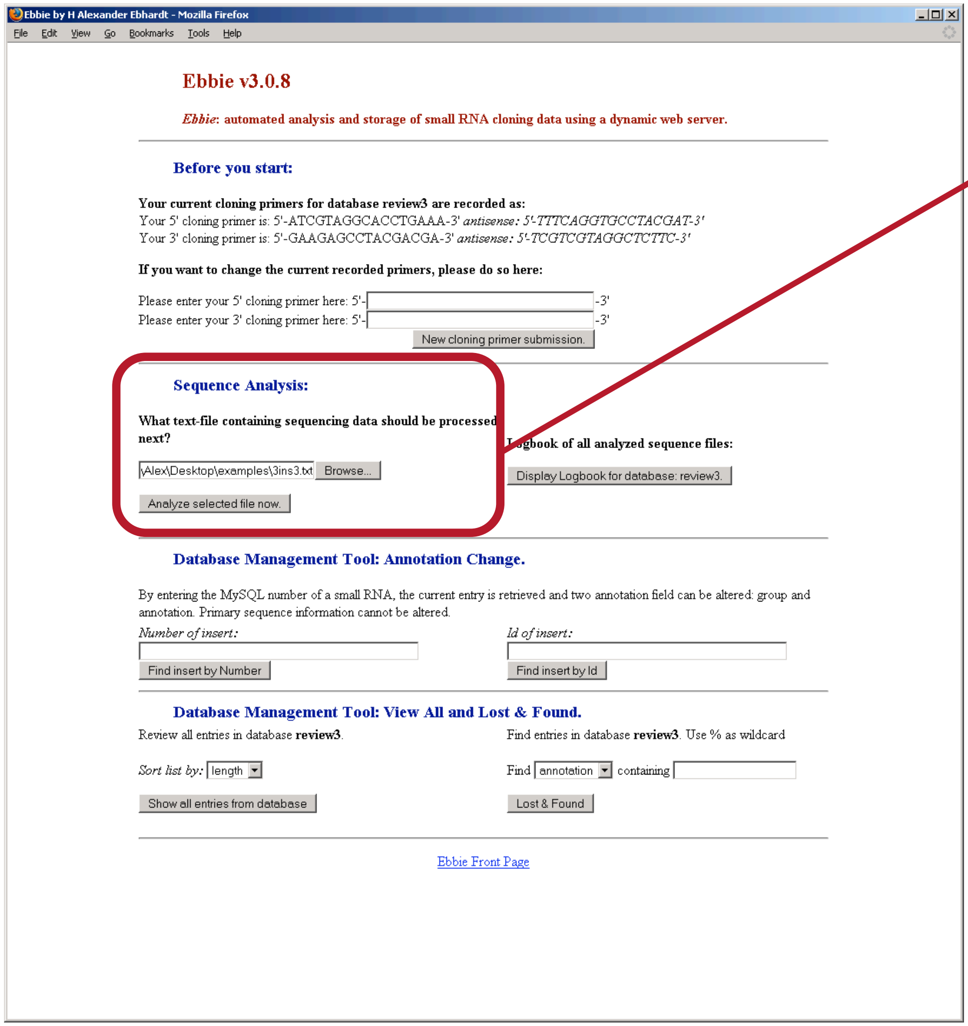

Main page of *Ebbie*: Multiple Inserts.

- Sequence Analysis:
  - Uploading text file containing DNA sequencing data

Ebbie v3.0.8

***Ebbie*: automated analysis and storage of small RNA cloning data using a dynamic web server.**

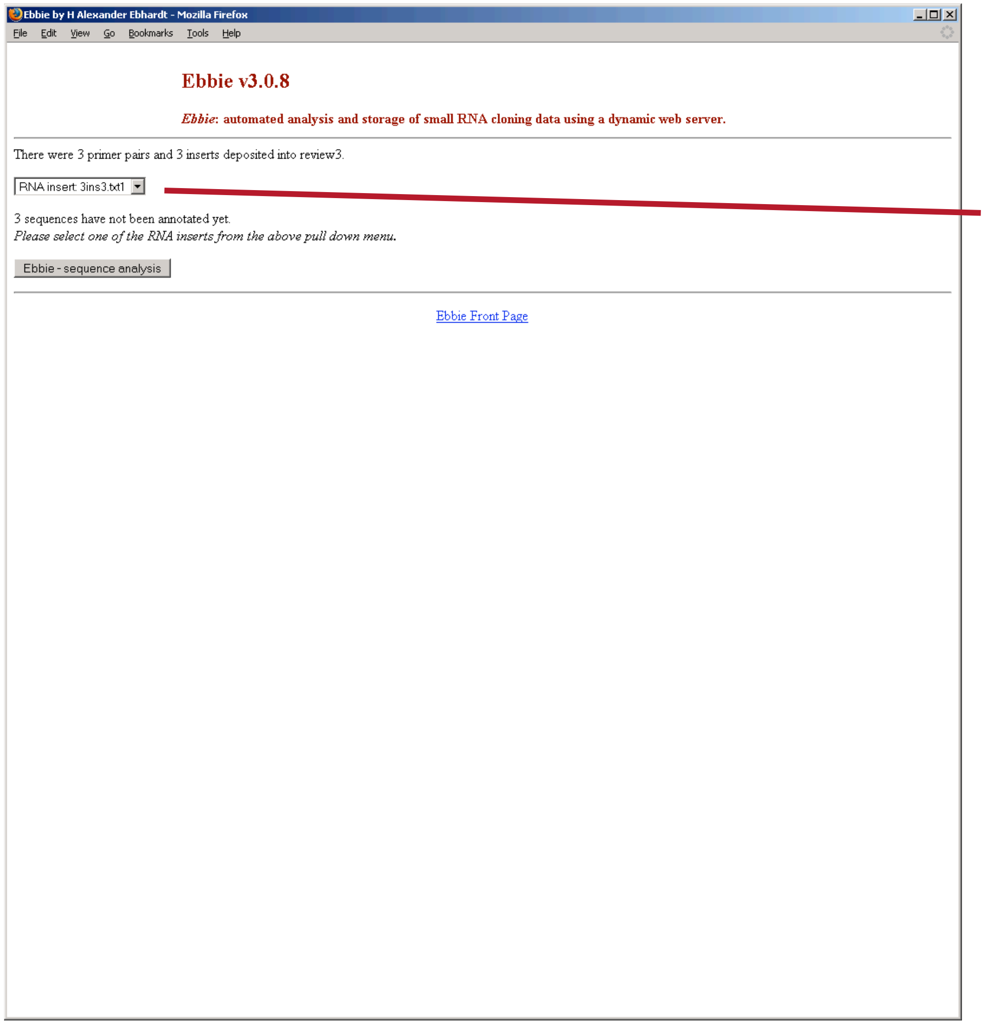

***Ebbie:* Multiple Inserts.**

- Program indicates how many primer pairs and how many inserts were found.
- From pull down menu, choose an insert.
- Press: 'Ebbie – sequence analysis submit button to start analysis of the insert.

Ebbie v3.0.8

Ebbie: automated analysis and storage of small RNA cloning data using a dynamic web server.

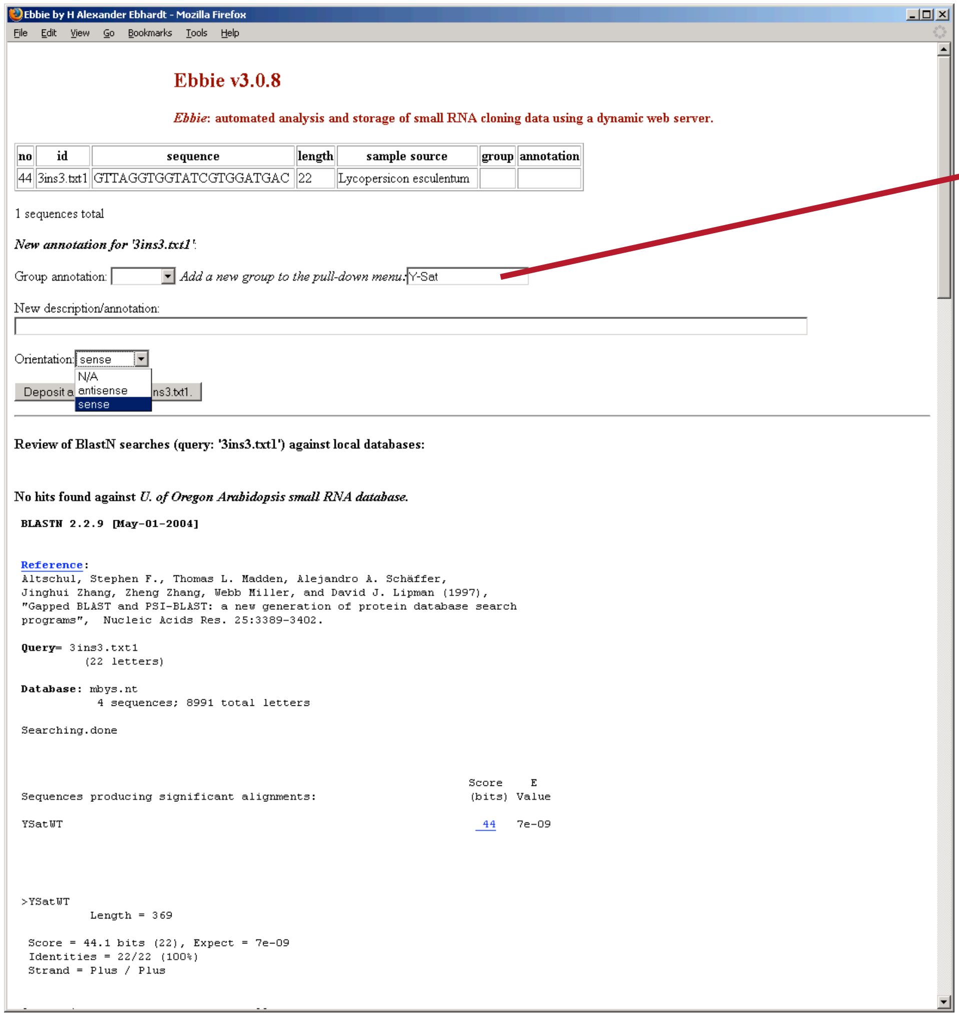

Ebbie: Multiple Inserts.

- BlastN analysis of insert
- Group annotation: added 'Y-Sat' to text menu.
- Deposit annotation…

Ebbie v3.0.8

***Ebbie*: automated analysis and storage of small RNA cloning data using a dynamic web server.**

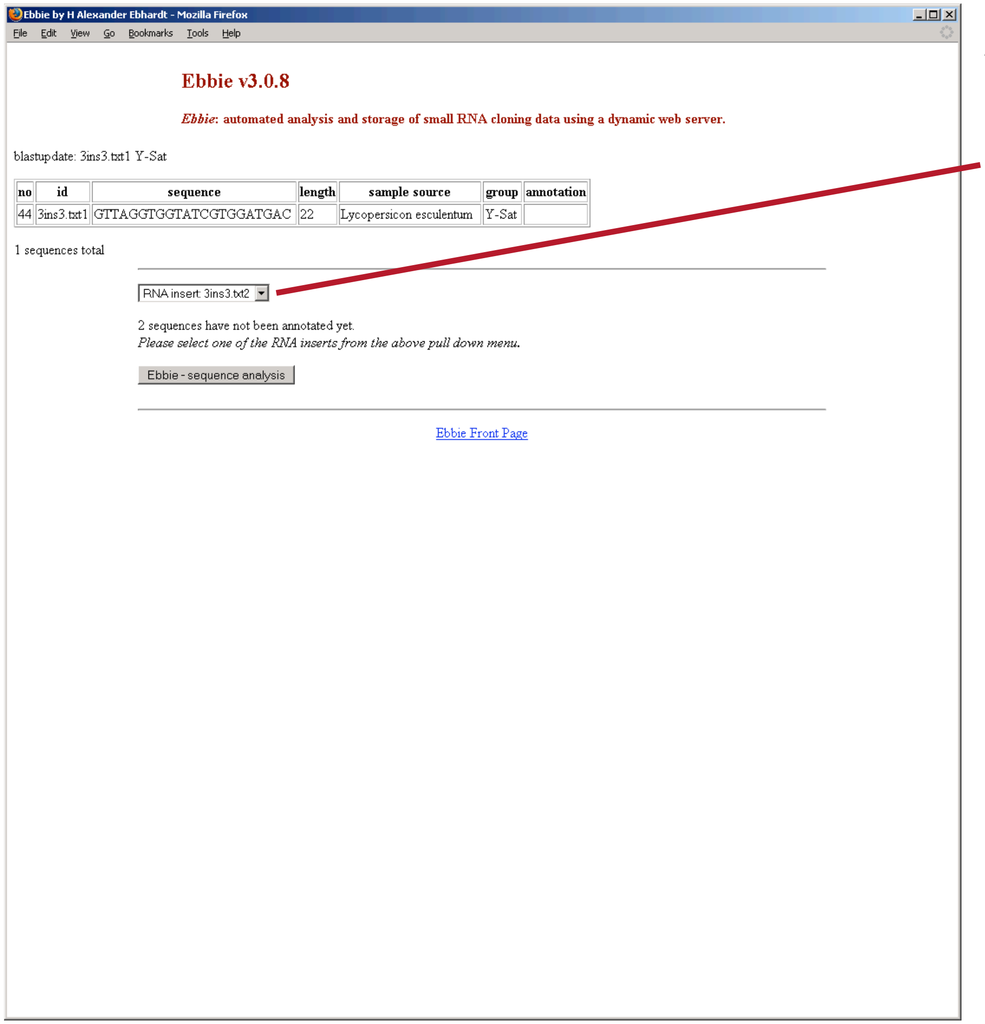

***Ebbie:* Multiple Inserts.**

- MySQL entry of previous insert depicted.
- Choose next insert from pull down menu (number of inserts remaining is shown below).
- Submit sequence.

Ebbie v3.0.8

Ebbie: automated analysis and storage of small RNA cloning data using a dynamic web server.

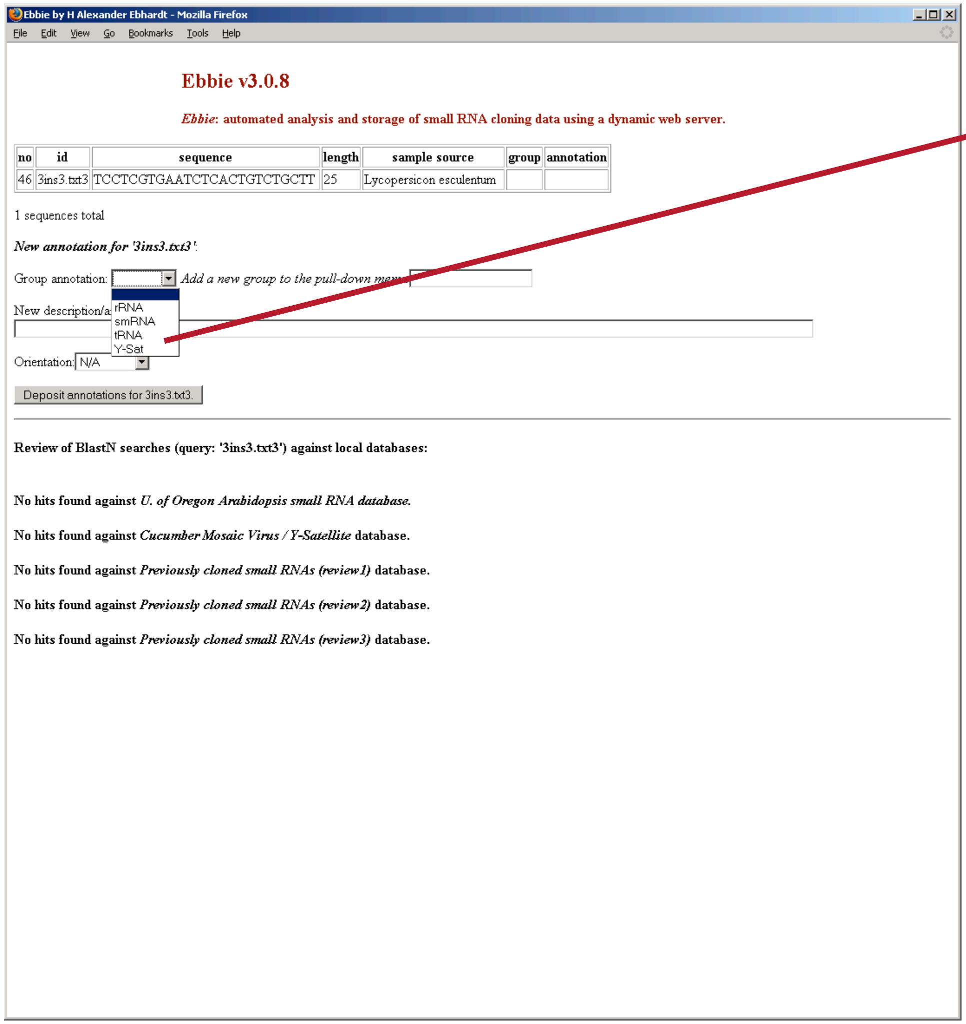

Ebbie: Multiple Inserts.

- 'Y-Sat' appears in alphabetical order (not case sensitive) in group pull down menu.

Ebbie v3.0.8

***Ebbie*: automated analysis and storage of small RNA cloning data using a dynamic web server.**

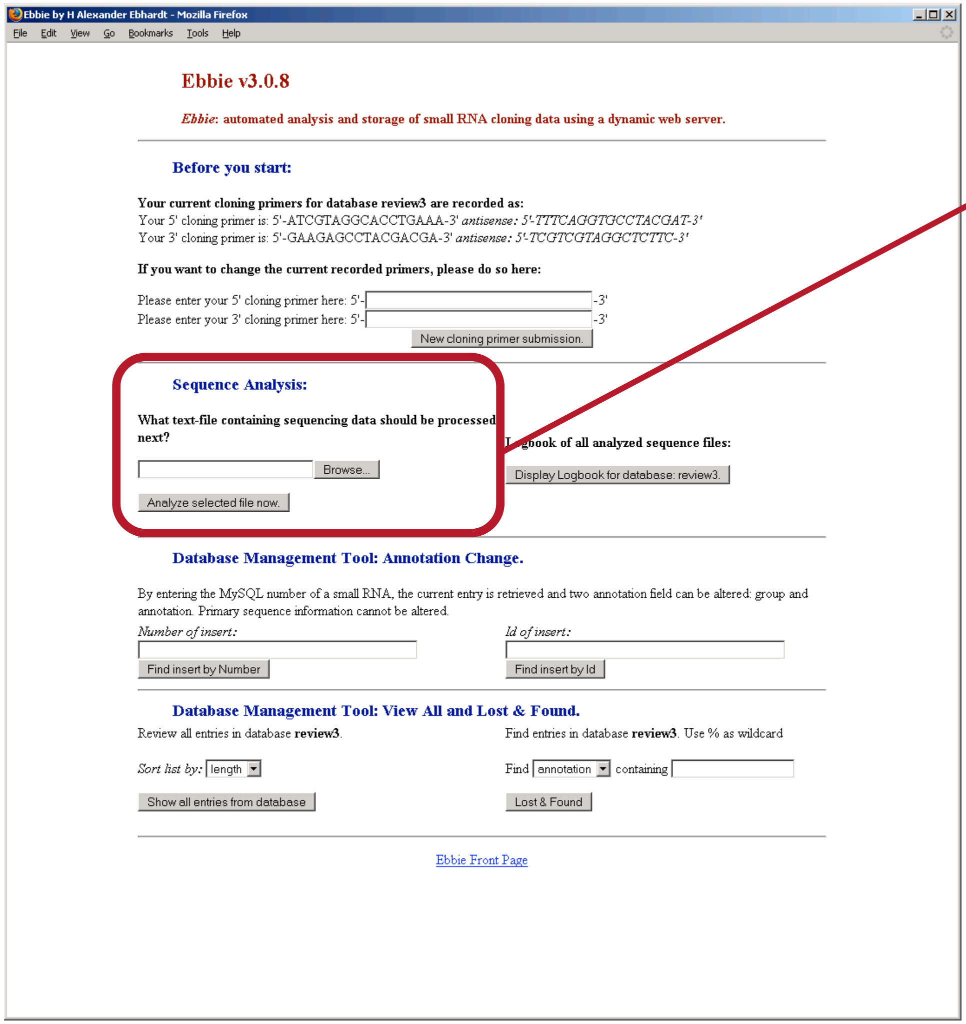

Ebbie: error messages:

- Sequence Analysis:
  - Uploading text file containing DNA sequencing data, error messages.

Ebbie v3.0.8

***Ebbie*: automated analysis and storage of small RNA cloning data using a dynamic web server.**

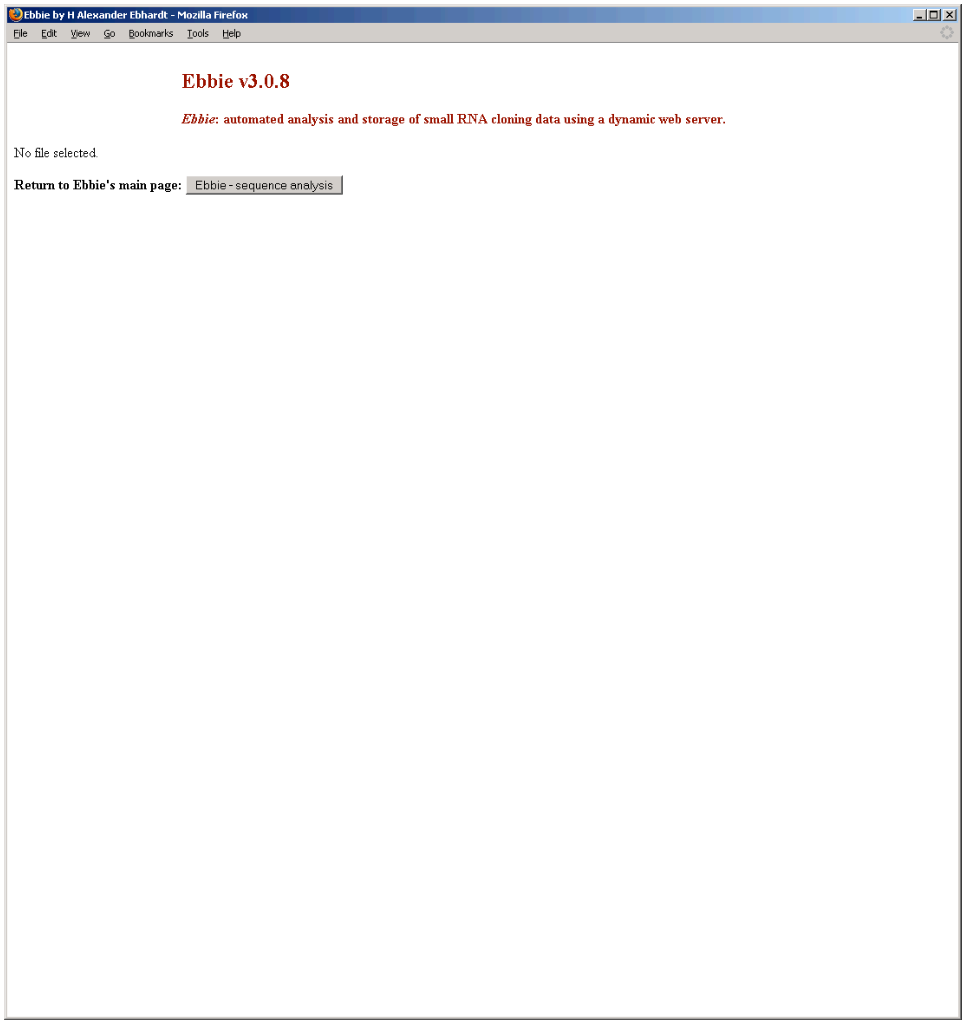

Ebbie: error messages:

- No file selected
- Filename already exists in database
- Uneven number of 5′- and 3′-cloning primer (logged*)
- No insert (logged*).

* logged: filename, date and comment will appear in logboook.

Ebbie v3.0.8

***Ebbie*: automated analysis and storage of small RNA cloning data using a dynamic web server.**

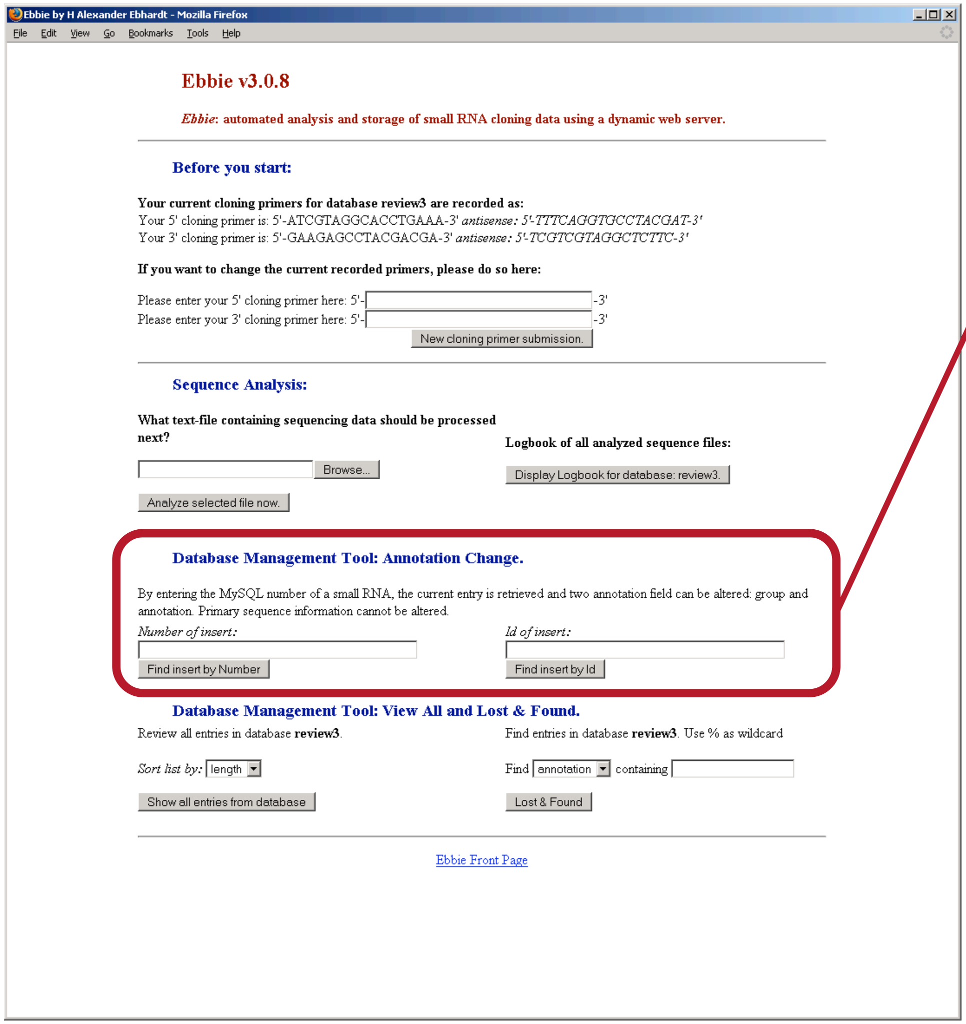

Ebbie: Database Management Tool: Annotation Change.

- Enter number or id of insert to update annotation.
  - For example, insert '44' into *Number of insert.*

Ebbie v3.0.8

***Ebbie*: automated analysis and storage of small RNA cloning data using a dynamic web server.**

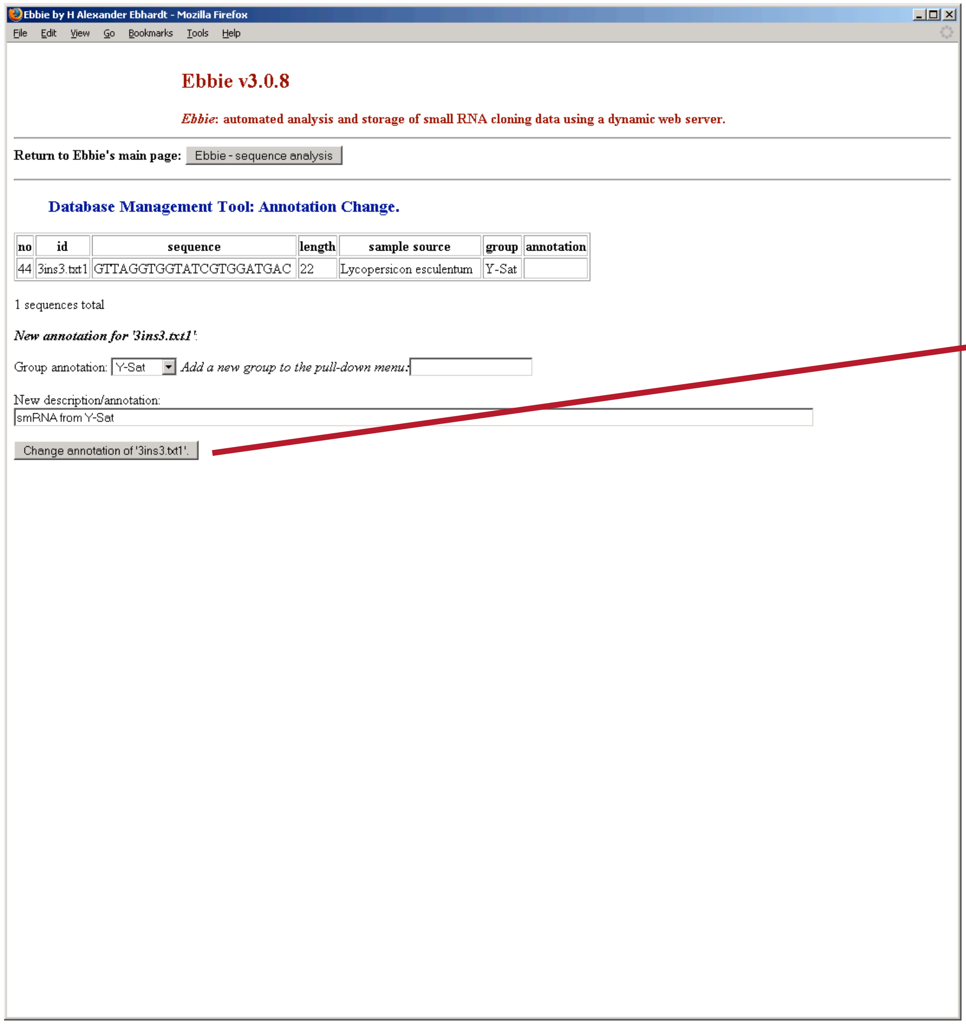

***Ebbie:* Database Management Tool: Annotation Change.**

- Database entry retrieved.
- Change group annotation.
- Update additional annotation.
- Submit updated information.

Ebbie v3.0.8

***Ebbie*: automated analysis and storage of small RNA cloning data using a dynamic web server.**

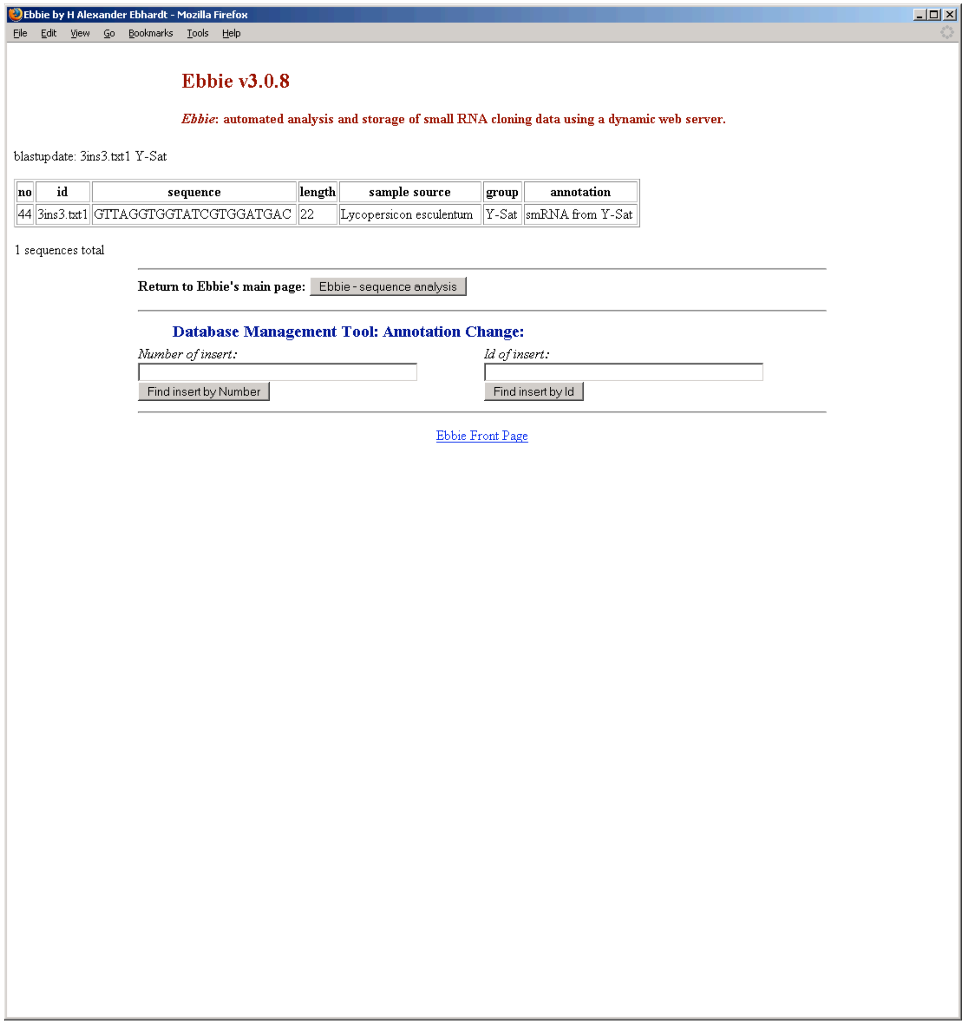

***Ebbie:* Database Management Tool: Annotation Change.**

- Updated information appears.
  - Go to *Ebbie*'s Main page.
  - Or enter id or number for annotation change tool.

Ebbie v3.0.8

Ebbie: automated analysis and storage of small RNA cloning data using a dynamic web server.

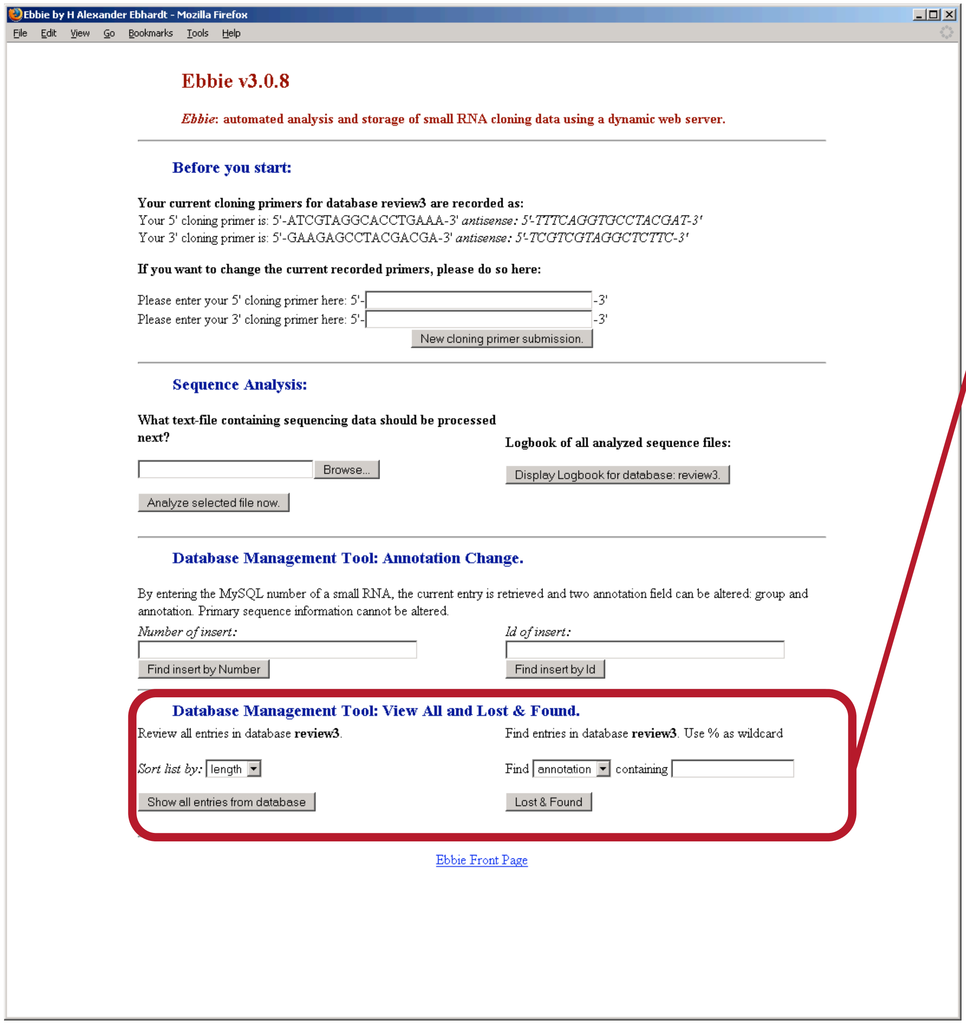

Main page of *Ebbie*.

- Database Management Tool: View All and Lost & Found
  - Review all entries, order by id, length, number or group.
  - Select a parameter and enter into text field a query using '%' as wild card character.

Ebbie v3.0.8

Ebbie: automated analysis and storage of small RNA cloning data using a dynamic web server.

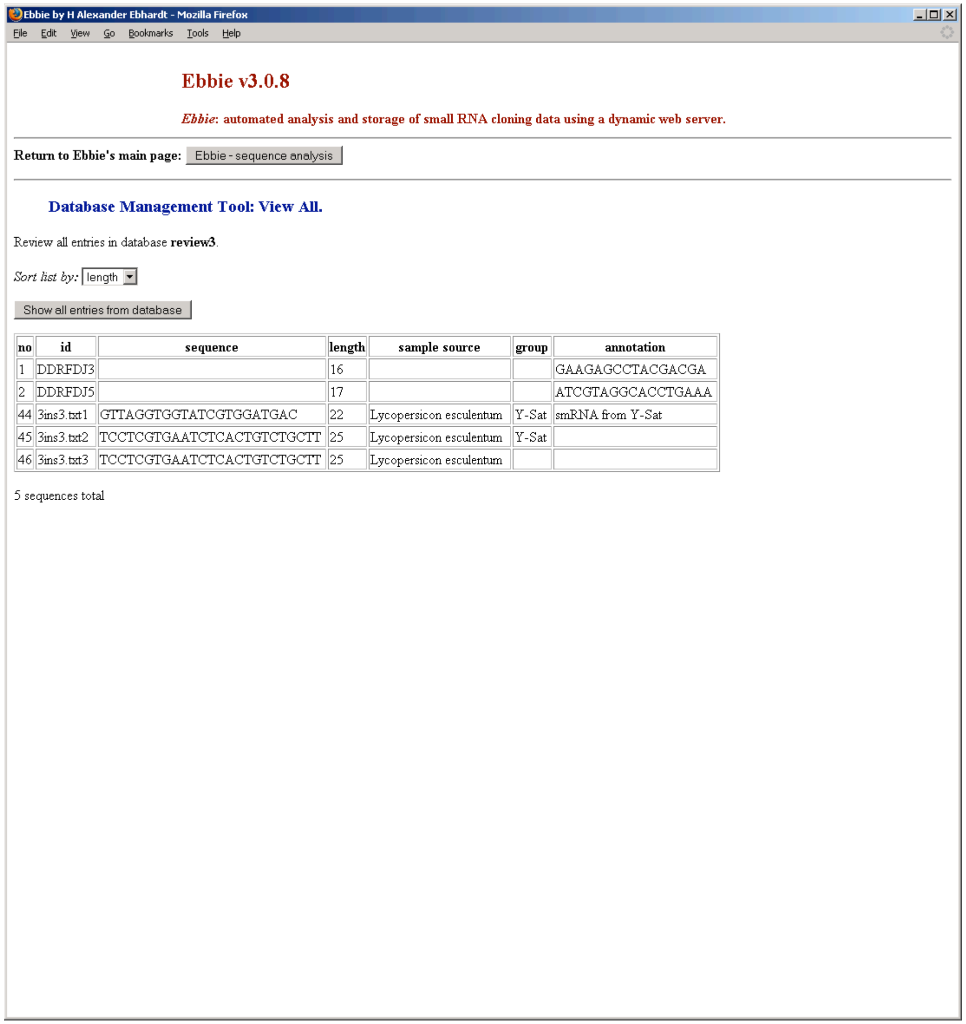

Ebbie by H Alexander Ebhardt - Mozilla Firefox

File Edit View Go Bookmarks Tools Help

Ebbie v3.0.8

Ebbie: automated analysis and storage of small RNA cloning data using a dynamic web server.

Return to Ebbie's main page: Ebbie - sequence analysis

Database Management Tool: View All.

Review all entries in database **review3**.

Sort list by: length

Show all entries from database

no	id	sequence	length	sample source	group	annotation
1	DDRFDJ3		16			GAAGAGCCTACGACGA
2	DDRFDJ5		17			ATCGTAGGCACCTGAAA
44	3ins3.txt1	GTTAGGTGGTATCGTGGATGAC	22	Lycopersicon esculentum	Y-Sat	smRNA from Y-Sat
45	3ins3.txt2	TCCTCGTGAATCTCACTGTCTGCTT	25	Lycopersicon esculentum	Y-Sat	
46	3ins3.txt3	TCCTCGTGAATCTCACTGTCTGCTT	25	Lycopersicon esculentum		

5 sequences total

Ebbie: **Database Management Tool: View All:**

- Sort by number, id, length and group.
- Displays all MySQL database entries.

Ebbie v3.0.8

Ebbie: automated analysis and storage of small RNA cloning data using a dynamic web server.

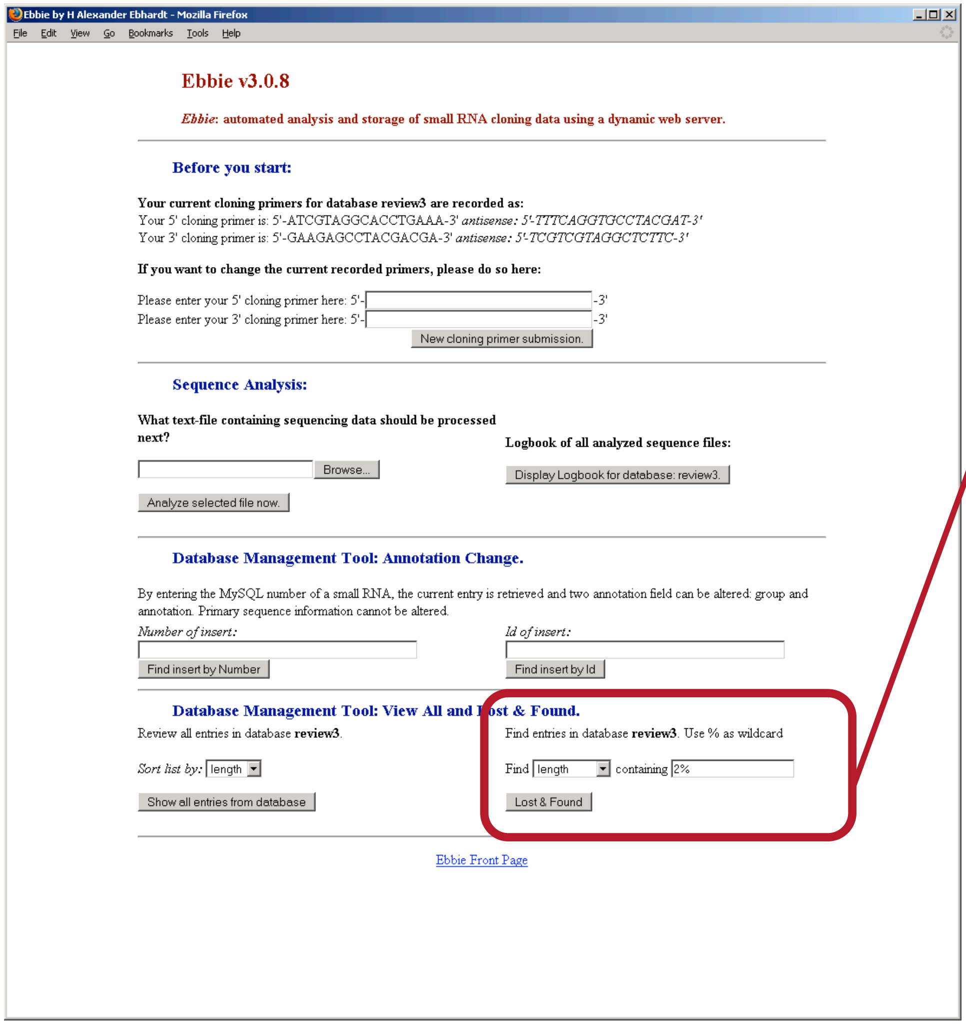

***Ebbie:* Database Management Tool: Lost & Found:**

– Enter query, e.g. length '2%'.

Ebbie v3.0.8

Ebbie: automated analysis and storage of small RNA cloning data using a dynamic web server.

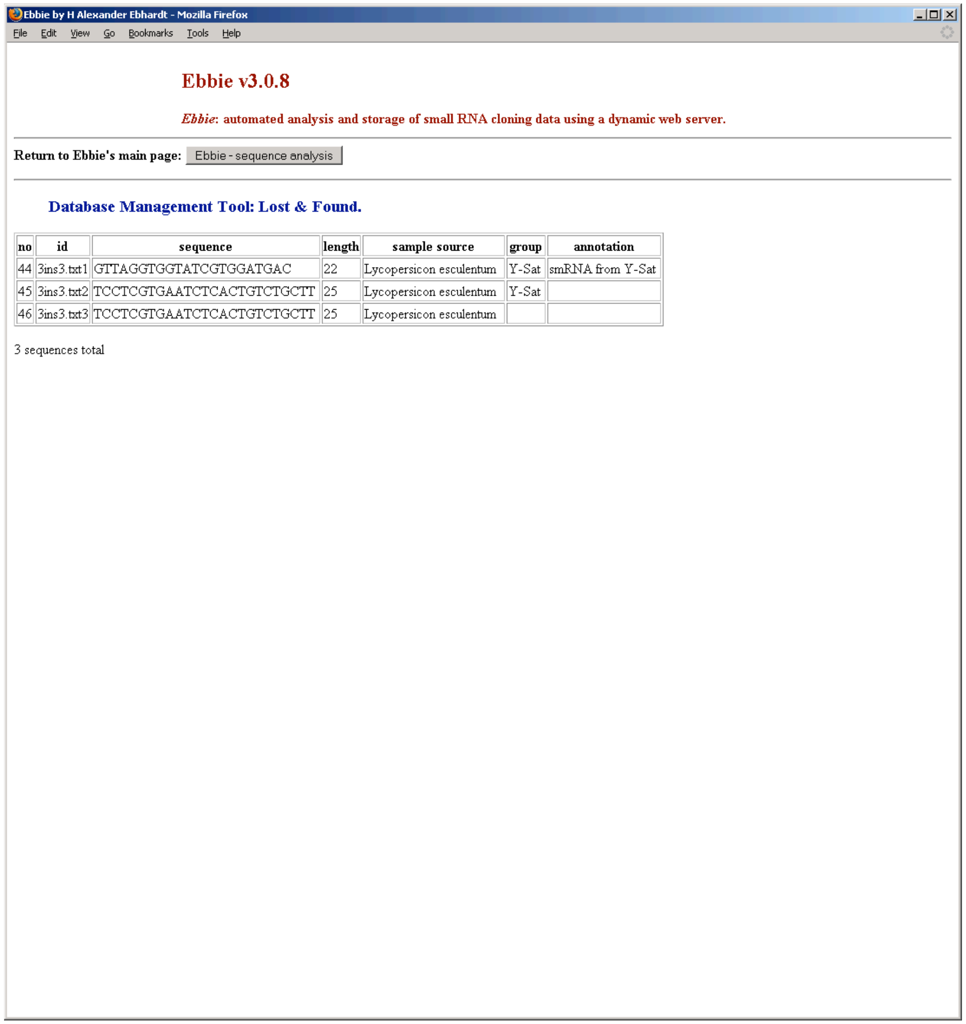
Ebbie by H Alexander Ebhardt - Mozilla Firefox

File Edit View Go Bookmarks Tools Help

Ebbie v3.0.8

Ebbie: automated analysis and storage of small RNA cloning data using a dynamic web server.

Return to Ebbie's main page: Ebbie - sequence analysis

Database Management Tool: Lost & Found.

no	id	sequence	length	sample source	group	annotation
44	3ins3.txt1	GTTAGGTGGTATCGTGGATGAC	22	Lycopersicon esculentum	Y-Sat	smRNA from Y-Sat
45	3ins3.txt2	TCCTCGTGAATCTCACTGTCTGCTT	25	Lycopersicon esculentum	Y-Sat	
46	3ins3.txt3	TCCTCGTGAATCTCACTGTCTGCTT	25	Lycopersicon esculentum		

3 sequences total

Ebbie: **Database Management Tool: Lost & Found:**

– Displays all MySQL database entries matching the query.

Ebbie v3.0.8

***Ebbie*: automated analysis and storage of small RNA cloning data using a dynamic web server.**

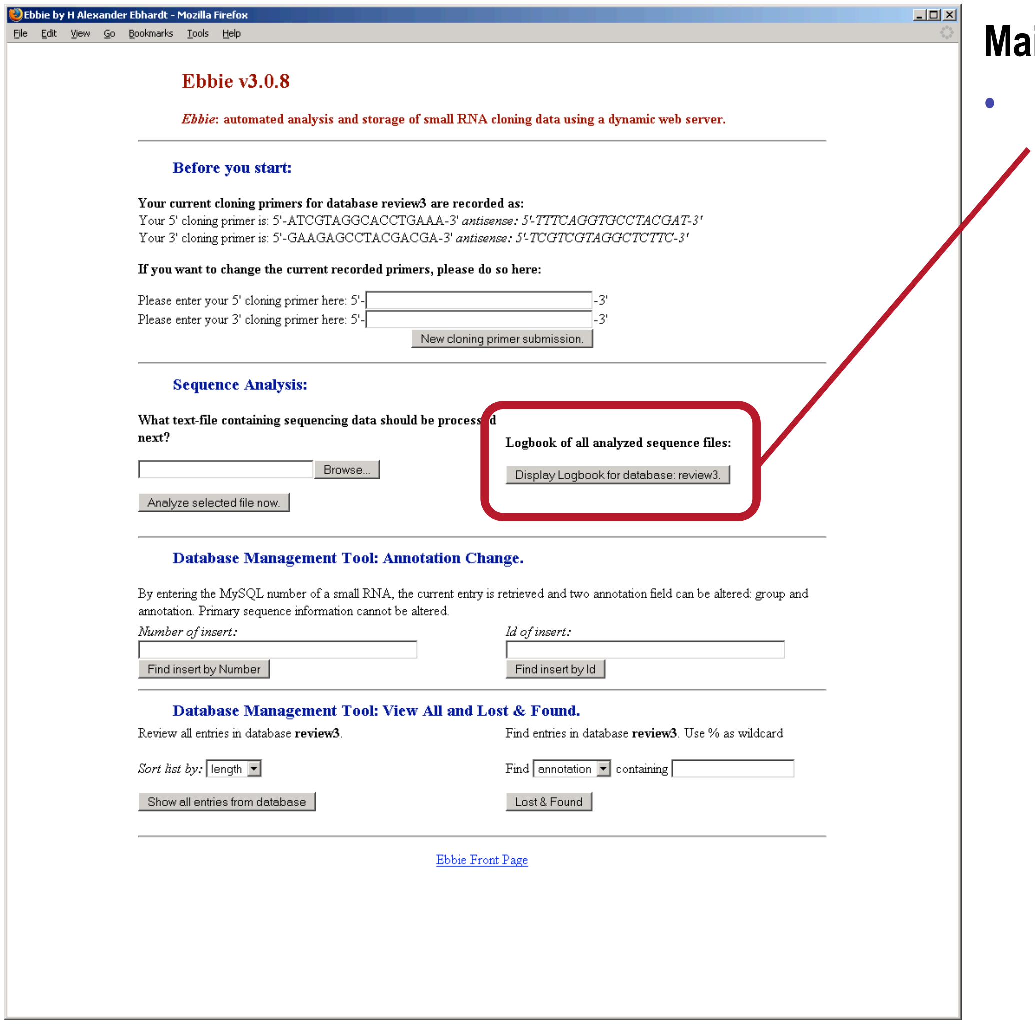

Main page of *Ebbie*.

- Sequence Analysis:
  - Logbook of all files analyzed.

Ebbie v3.0.8

***Ebbie*: automated analysis and storage of small RNA cloning data using a dynamic web server.**

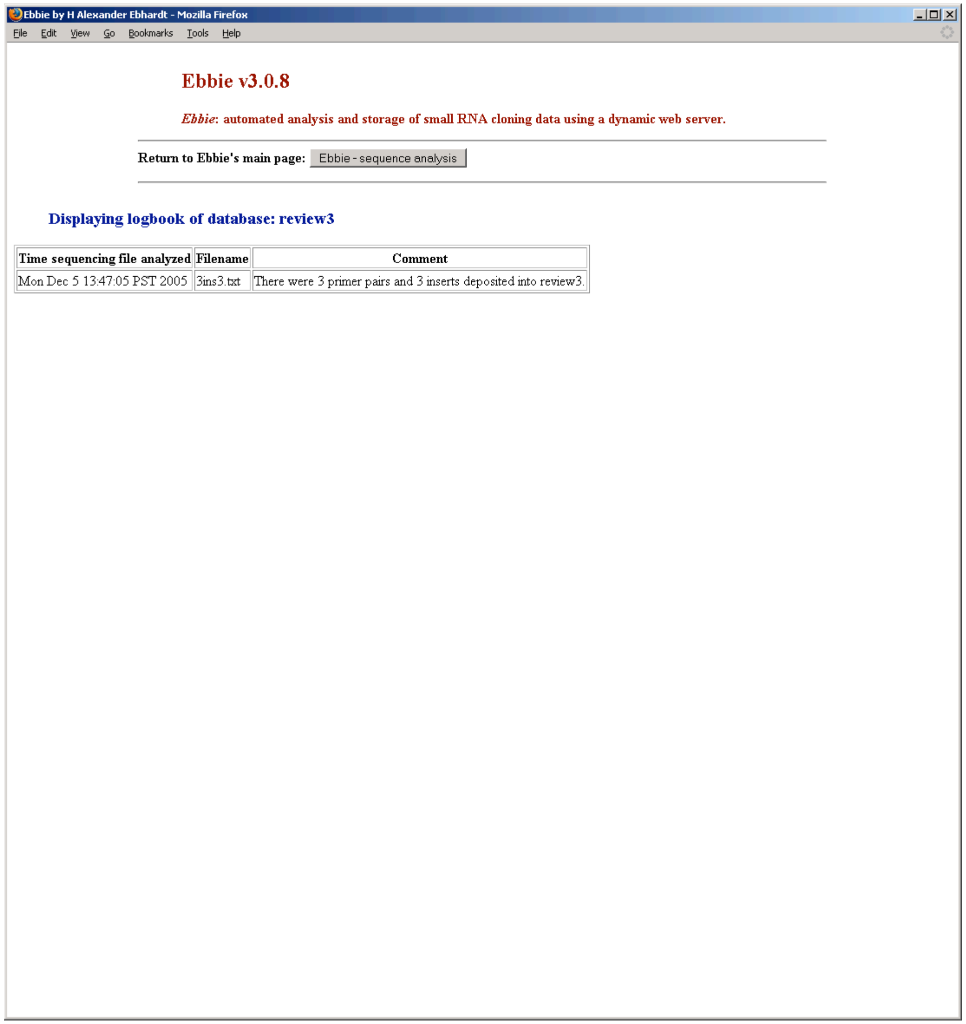

Ebbie: Logbook

- Displays all files analyzed.
- Table: date (system time), filename and comment.
- Comments are:
  - Single insert found.
  - Number of 5'- and 3'- cloning primers uneven!
  - There were *x* primer pairs and *y* inserts deposited into *z* (where *x* is the number of inserts / smRNAs found, *y* the number of primer pairs and *z* the database).
  - Single insert found.

End of Tutorial.
